# STAT3 associates with vacuolar H^+^-ATPase and regulates cytosolic and lysosomal pH

**DOI:** 10.1038/s41422-018-0080-0

**Published:** 2018-08-20

**Authors:** Bin Liu, Johan Palmfeldt, Lin Lin, Alexandria Colaço, Knut K. B. Clemmensen, Jinrong Huang, Fengping Xu, Xin Liu, Kenji Maeda, Yonglun Luo, Marja Jäättelä

**Affiliations:** 1 Cell Death and Metabolism, Center for Autophagy, Recycling and Disease (CARD), Danish Cancer Society Research Center (DCRC), DK-2100 Copenhagen, Denmark; 2 Research Unit for Molecular Medicine, Department of Clinical Medicine, Aarhus University Hospital and Faculty of Health, Aarhus University, DK-8200 Aarhus, Denmark; 3 Department of Biomedicine, Aarhus University, DK-8000 Aarhus, Denmark; 4 BGI-Shenzhen, Shenzhen, Guangdong 518083 China; 5 Department of Biology, University of Copenhagen, DK-2200 Copenhagen, Denmark; 6 BGI-Qingdao, Qingdao, Shandong 266555 China; 7 Department of Cellular and Molecular Medicine, Faculty of Health Sciences, University of Copenhagen, DK-2200 Copenhagen, Denmark

## Abstract

Dysregulated intracellular pH is emerging as a hallmark of cancer. In spite of their acidic environment and increased acid production, cancer cells maintain alkaline intracellular pH that promotes cancer progression by inhibiting apoptosis and increasing glycolysis, cell growth, migration, and invasion. Here we identify signal transducer and activator of transcription-3 (STAT3) as a key factor in the preservation of alkaline cytosol. STAT3 associates with the vacuolar H^+^-ATPase in a coiled-coil domain-dependent manner and increases its activity in living cells and in vitro. Accordingly, STAT3 depletion disrupts intracellular proton equilibrium by decreasing cytosolic pH and increasing lysosomal pH, respectively. This dysregulation can be reverted by reconstitution with wild-type STAT3 or STAT3 mutants unable to activate target genes (Tyr705Phe and DNA-binding mutant) or to regulate mitochondrial respiration (Ser727Ala). Upon cytosolic acidification, STAT3 is transcriptionally inactivated and further recruited to lysosomal membranes to reestablish intracellular proton equilibrium. These data reveal STAT3 as a regulator of intracellular pH and, vice versa, intracellular pH as a regulator of STAT3 localization and activity.

## INTRODUCTION

Tumorigenesis proceeds via an evolutionary process, in which a succession of genetic changes provide the transforming cells with a set of acquired capabilities that enable tumor growth and dissemination.^1^ These traits include sustained proliferative signaling, metastatic capacity, activation of angiogenesis, replicative immortality, reprogrammed energy metabolism, as well as escape from cell death, growth suppressors, and immune destruction. Besides these well-established hallmarks of cancer, the pH gradient reversal, i.e., acidification of extracellular pH (pH_e_) from ∼7.4 in normal cells to 6.5–7.0 in cancer cells, while maintaining alkaline cytosolic pH (pH_c_) of normal cells (∼7.2) or further alkalizing it to values as high as 7.6 in cancer cells, is emerging as a universal hallmark of cancer observed in malignant tumors regardless of the pathology, genetics, and origin.^2–4^ The reversal of the pH gradient is an early event in tumorigenesis and its maintenance reinforces metabolic adaptation, tumor cell survival, invasion, immune evasion, and drug resistance. For instance, glycolytic flux essential for metabolic reprogramming is stimulated by alkaline cytosol,^3^ whereas the activation of apoptosis-inducing caspases depends on mild acidification of the cytosol.^5^ In parallel, the acidification of the extracellular space promotes tumor immune escape and effective proteolytic degradation of extracellular matrix by invading tumor cells.^6,7^ Thus, in line with genome instability, pH gradient reversal could be considered as an underlying cellular requirement for acquiring and maintaining several other cancer traits during tumorigenesis. Yet, our knowledge of its formation and maintenance is rather rudimentary. Hitherto, plasma membrane-localized ion transporters, including Na^+^/H^+^ exchanger 1 (NHE1), proton-linked monocarboxylate transporters and vacuolar H^+^-ATPase (V-ATPase), as well as carbonic anhydrases, have been identified as proteins contributing to the cancer-associated increase in net acid extrusion.^3^ In addition to the acid removal via the plasma membrane, V-ATPase pumps protons from the cytosol into intracellular vesicles of the endo-lysosomal compartment, especially late endosomes and lysosomes, which serve as major intracellular proton stores.^8–10^ For simplicity, we hereafter refer to all organelles detected by fluorescent dextran loading or staining for V-ATPase subunits or lysosome-associated membrane proteins LAMP1 or LAMP2 as lysosomes. Compared with normal cells, most invasive cancer cells have an enlarged and highly acidic lysosomal compartment, more peripherally localized lysosomes, and an increase in lysosomal exocytosis.^11–13^ Thus, the lysosomal V-ATPase may contribute to the establishment and maintenance of the reversed pH gradient of cancer cells by removing cytosolic protons to the lysosomal lumen, from where they can be effectively discarded to the extracellular space via lysosomal exocytosis.

V-ATPase is a large multi-subunit complex composed of 14 different proteins that are organized into a water soluble, ATP-hydrolyzing V_1_ domain, and a membrane-embedded V_o_ proton channel, which function together by coupling the energy of ATP hydrolysis to the transport of protons across the lipid bilayer.^8–10^ The V-ATPase-mediated acidification of lysosomal lumen is essential not only for the cargo degradation but also for the cellular metabolism in general, e.g., through the regulation of several key signaling pathways, including mechanistic target of rapamycin complex 1 and Notch pathways.^10,14^ Furthermore, V-ATPase activity has an important role in cancer cells by enhancing their metastatic potential, chemotherapy resistance, and survival in the acidic tumor environment.^15–17^

Signal transducer and activator of transcription-3 (STAT3) was originally identified as a latent cytosolic transcription factor, which could be activated by interferons and related cytokines to drive the expression of acute phase genes regulating inflammation and immunity.^18^ Today, STAT3 is known as a pleiotropic transcription factor that is commonly activated in various cancers, where it can act as an oncogene by activating genes involved in differentiation, proliferation, apoptosis, metastasis, angiogenesis, and metabolism.^19–22^ It is a member of STAT protein family that consists of seven structurally related members in mammals.^23,24^ Akin to other STATs, STAT3 contains six well-defined structural domains as follows: NH_2_-terminal domain, coiled-coil domain, DNA-binding domain, linker domain, Src homology 2 (SH2) domain, and transcriptional activation domain.^25,26^ Its transcriptional activity depends on the phosphorylation of Tyr705 in the transcriptional activation domain. Phosphorylated STAT3 can form homodimers or heterodimers with other STAT proteins via reciprocal SH2-phosphotyrosine interactions and translocate into the nucleus to activate gene transcription. In normal cells, the transcriptional activation occurs rapidly in response to cytokine signaling and is transient, whereas oncogenes with tyrosine kinase activity, e.g., c-Src and activated members of epidermal growth factor receptor family, keep STAT3 constitutively active in many cancers.^27–29^ For a long time, it has been assumed that STAT3-mediated promotion of tumor growth depends entirely on its well-described transcriptional activities. STAT3 has, however, been demonstrated to control cell metabolism and migration in a transcription-independent manner. A small pool of acetylated and Ser727-phosphorylated STAT3 is localized to mitochondria, where it may promote malignant transformation and cancer progression by supporting the optimal function of mitochondrial electron transport chain and inhibiting the production of reactive oxygen species.^30–32^ Furthermore, association of cytoplasmic STAT3 with a mictotublule-destabilizing protein stathmin potentiates microtubule polymerization and cell movement.^33^ Thus, transcriptional and cytoplasmic activities of STAT3 may function in concert to promote tumorigenesis.

Inspired by our finding of lysosome-associated STAT3, we investigated its role in intracellular pH regulation. Here we demonstrate that STAT3 associates with the lysosomal V-ATPase complex, stimulates its ATPase activity, and contributes to the maintenance of the alkaline cytosol and acidic lysosomal lumen.

## RESULTS

### STAT3 localizes to the lysosomal membrane

**Fig. 1:**
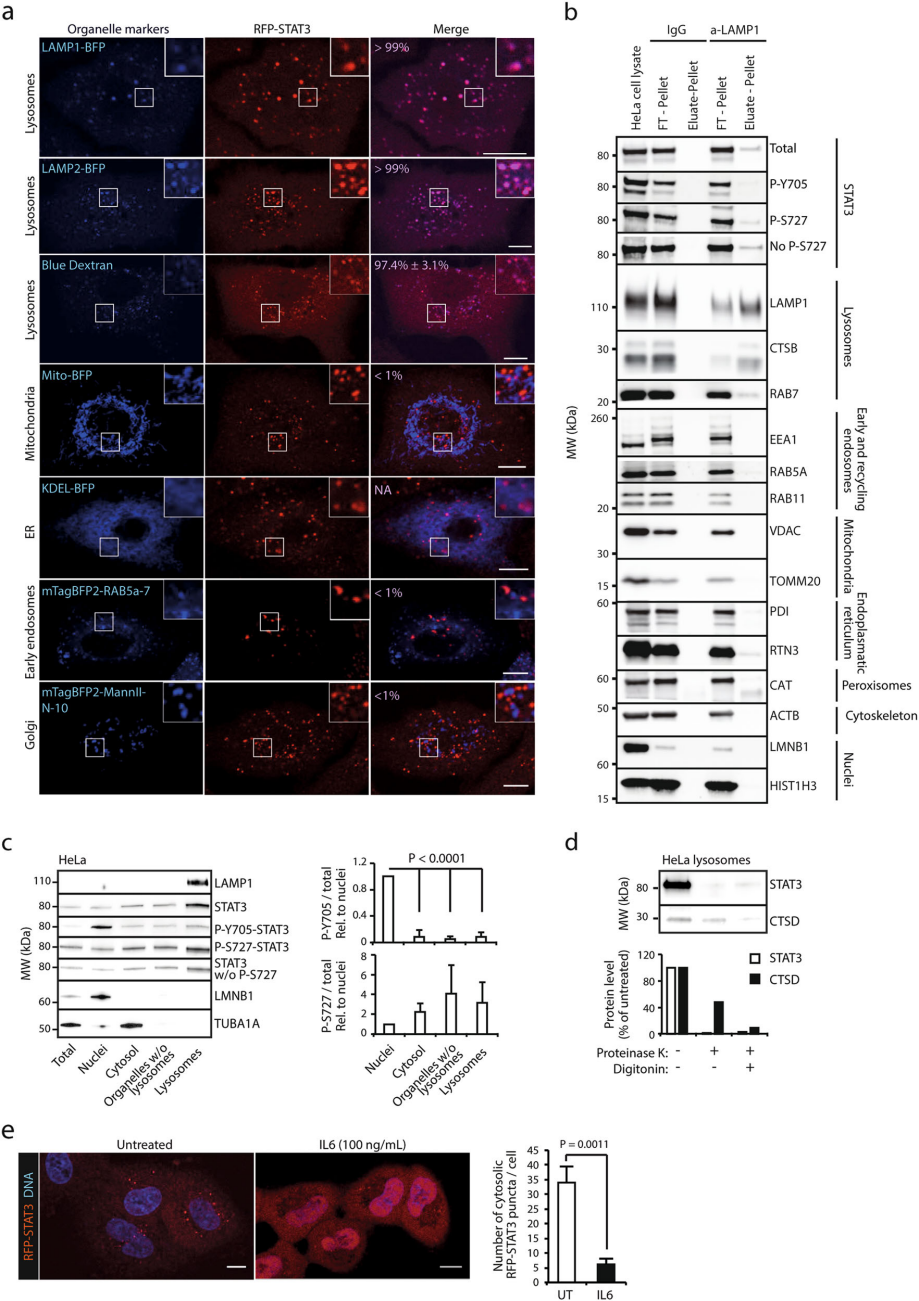
STAT3 localizes to the lysosomal membrane. **a** Representative images of A549-RFP-STAT3 cells labeled with the indicated organelle markers (blue). Values, mean percentage of RFP-STAT3 puncta colocalizing with the indicated organelle marker ± SD of three independent experiments with ≥ 10 cells/sample analyzed in each. Colocalization analysis was not applicable (NA) in KDEL-BFP-labeled cells due to the diffuse staining. The areas marked with white squares are magnified in upper right corners. Images of live cells were taken with 60× magnification using Zeiss LSM700 confocal microscope. See Supplementary information, Fig. S1 for colocalization of STAT3 and lysosomes in other cells. **b** Representative immunoblots of STAT3 and the indicated organelle markers in total cell lysates or the indicated flow throughs (FT) and immunoprecipitates (eluate) from HeLa cells. *n* = 3. **c** Representative immunoblots of the indicated proteins in total cell lysates or the indicated fractions of HeLa cells (left) and quantification of P-Y705-STAT3 and P-S727-STAT3 levels relative to total STAT3. **d** Representative immunoblots (top) and quantification (bottom) of the indicated proteins in lysates of HeLa cell lysosomes purified by iron-dextran method and left untreated or treated with 10 µg/ml proteinase K and 100 µg/ml digitonin for 10 min at 25 °C when indicated. CTSD, cathepsin D. **e** Representative images (left) and quantification of cytosolic RFP-STAT3 puncta (right) in A549-RFP-STAT3 cells left untreated or treated with 100 ng/mL IL6 for 30 min and stained with Hoechst. Error bars, SD of three independent experiments, with ≥ 10 cells analyzed/sample. *P*- values were calculated by one-way ANOVA combined with Dunnett’s multiple comparisons test (**c**) or two-tailed, homoscedastic Student’s *t*-test (**e**). The optimal slice thickness (∼350 nm) of confocal images (**a**, **e**) was defined by the Zeiss zen software. Scale bar, 10 µm

Prompted by the punctate lysosome-like pattern of RFP-STAT3 in live A549 non-small cell lung cancer cells, in which the NH_2_ terminus of the endogenous *STAT3* gene is tagged with a red fluorescent protein (RFP) using transcription activator-like effector nuclease-mediated knock-in,^34^ we investigated the putative lysosomal localization and function of STAT3. For this purpose, we took advantage of the above-mentioned A549-RFP-STAT3 cells and confirmed that in addition to a diffuse cytoplasmic localization, RFP-tagged endogenous STAT3 formed numerous cytoplasmic puncta in untreated cells (Fig. 1a). Labeling the organelles of A549-RFP-STAT3 cells with appropriate fluorescent markers revealed that the majority of the RFP-STAT3 puncta colocalized with lysosomes labeled with cascade blue dextran or blue fluorescent protein (BFP) fused to either LAMP1 or LAMP2, whereas no significant colocalization was observed between RFP-STAT3 puncta and markers of mitochondria, early endosomes, or Golgi apparatus (Fig. 1a). The marker for endoplasmatic reticulum stained large parts of the cytoplasm and therefore the resolution of confocal microscopy did not allow a proper analysis of its colocalization with RFP-STAT3 (Fig. 1a). To test whether endogenous STAT3 localized to lysosomes in other cell types, we generated SKOV3 ovarian carcinoma cells with enhanced green fluorescent protein (EGFP) fused to the NH_2_ terminus of the endogenous *STAT3* gene using CRISPR-Cas9 gene editing. Akin to A549 cells, EGFP-tagged endogenous STAT3 formed cytoplasmic puncta that colocalized with lysosomes in live SKOV3 cells (Supplementary information, Fig. S1a). Notably, the punctate pattern and lysosomal localization of STAT3 in A549-RFP-STAT3 and SKOV3-EGFP-STAT3 cells was largely abolished by treatments commonly used to permeabilize fixed cells for immunocytochemistry, i.e., methanol, saponin and triton (Supplementary information, Fig. S1b and data not shown), which may explain why the abundant lysosomal localization of STAT3 has not been discovered earlier. Accordingly, co-staining of the endogenous, non-tagged STAT3 and ATP6V01D, a lysosomally localized subunit of V-ATPase, in fixed and saponin-permeabilized HeLa cervix cancer cells showed less colocalization of STAT3 and lysosomes than observed by live-cell imaging (Supplementary information, Fig. S1c). Confirming its association with HeLa cell lysosomes, ∼5% of total cellular STAT3 protein associated with lysosomes immunopurified with antibodies against the cytosolic tail of LAMP1 or captured with a magnet after iron-dextran loading of the lysosomal compartment (Fig. 1b, c).

Although the nuclear and mitochondrial localizations of STAT3 depend on the phosphorylation of Tyr705 and Ser727, respectively, the phosphorylation status of these sites in lysosomal STAT3 did not show clear preference to either modification (Fig. 1b, c). To find out on which side of the lysosomal membrane STAT3 resided, we exposed intact lysosomes to proteinase K in the presence or the absence of a digitonin detergent. Unlike luminal cathepsin D protein, which was effectively digested by proteinase K only in the presence of detergent, STAT3 was completely digested in the absence of detergent (Fig. 1d). These data suggest that STAT3 resides on the cytosolic surface of the lysosomal membrane and should therefore be able to be mobilized by an appropriate stimulus. Correspondingly, treatment of A549-RFP-STAT3 cells with interleukin 6, a potent inducer of nuclear localization and transcriptional activity of STAT3,^35^ triggered a rapid decline in cytoplasmic STAT3-positive puncta and a simultaneous accumulation of STAT3 in nuclei (Fig. 1e). Taken together, these data suggest that in the absence of activating stimuli, a fraction of cellular STAT3 is loosely attached to the cytosolic side of lysosomal limiting membrane.

### STAT3 interacts with V-ATPase on the lysosomal membrane via its coiled-coil domain

**Fig. 2:**
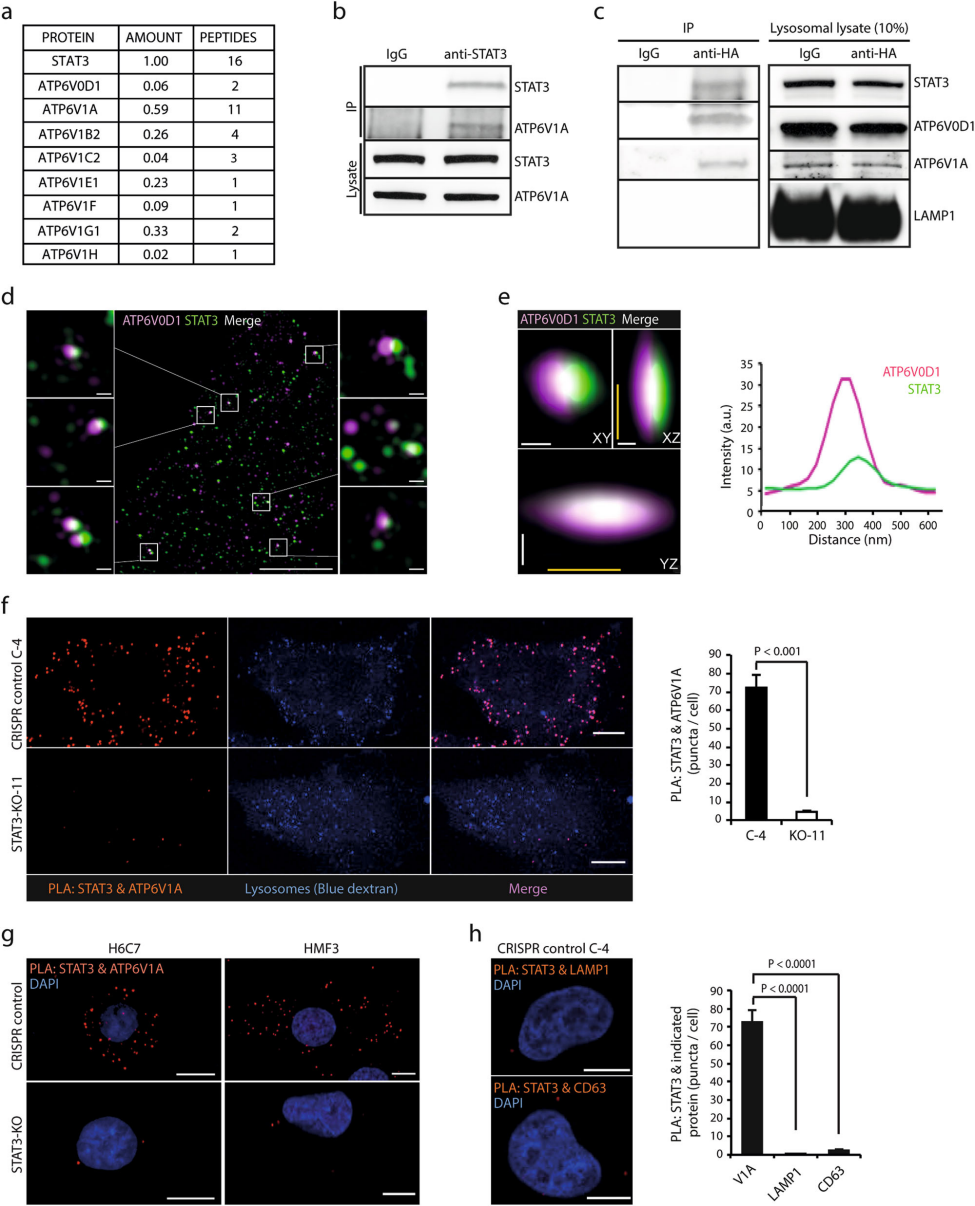
STAT3 interacts with V-ATPase on the lysosomal membrane. **a** V-ATPase subunits detected by high-accuracy mass spectrometry in anti-Flag immunoprecipitates of lysosomal lysates from HeLa cells transiently transfected with pBCMV-STAT3-Flag-puro. Protein amount in relation to STAT3 was estimated from summed peptide intensities (iBAQ algorithm in Maxquant software). The criteria for false discovery rate (FDR) of peptide spectrum match was set to 0.05 and protein FDR to 0.01. Numbers of peptides with identification *P*-values < 0.01 are indicated. Experiment was repeated once with similar results. **b** Representative immunoblots of endogenous STAT3 and ATP6V1A (V1A) in anti-STAT3 immunoprecipitates (IP) and lysates of crude lysosome fractions of HeLa cells. *n* = 3. **c** Representative immunoblots of the indicated proteins in anti-HA immunoprecipitates (IP) and lysates of crude lysosome fractions of HeLa cells transiently transfected with pCDNA3.1-HA-ATP6V1A. *n* = 3. **d** A representative SR-SIM image of a HeLa cell stained with anti-STAT3 and anti-ATP6V0D1 antibodies. The areas marked with white squares are magnified in both sides. Scale bars, 5 µm (middle) and 200 nm (left and right). **e** Representative images of the indicated SR-SIM projections (left) and colocalization analysis (right) of a punctum staining with anti-STAT3 and anti-ATP6V0D1 (V0D1) antibodies in HeLa cells. Scale bars, 145 nm (yellow) and 200 nm (white). **f** Representative images (left) and quantification (right) of PLA puncta with antibodies against STAT3 and ATP6V1A in HeLa CRISPR control cell clone (C-4) and STAT3-KO clone (STAT3-KO-11). Lysosomes were visualized by loading with cascade blue dextran. See Fig. 4a for the immunoblot verifying STAT3 depletion. **g** Representative images of PLAs using antibodies against STAT3 and ATP6V1A in CRISPR control and STAT3-KO human pancreatic duct epithelial cells (H6C7) and human mammary fibroblasts (HMF3) counterstained with DAPI. See Supplementary information, Fig. S2c for the immunoblot verifying STAT3 depletion. **h** Representative images (left) and quantification (right) of PLA puncta with antibodies against STAT3 and LAMP1 or CD63 in HeLa-C-4 clone. Nuclei were counterstained with DAPI. Cell images in **f**–**h** were taken with 60×  magnification using Zeiss LSM700 confocal microscope. The optimal slice thickness (∼350 nm) was defined by the Zeiss zen software. The images of H6C7 cells in **g** are integrated stacks. Scale bar, 10 µm. Error bars, SD of three independent experiments with ≥ 10 cells analyzed/sample. *P*-values were calculated by two-tailed, homoscedastic Student’s *t*-test (**f**) or one-way ANOVA combined with Dunnett’s multiple comparisons test (**h**)

To understand the putative function of lysosomal STAT3, we searched for STAT3-interacting proteins in lysosomal extracts of HeLa cells transiently transfected with Flag-tagged STAT3 (STAT3-Flag). Nanoscale-liquid chromatography tandem mass spectrometry (nano-LC-MS/MS) analysis of proteins co-immunoprecipitating with STAT3-Flag identified eight subunits of the V-ATPase complex as potential STAT3-binding partners (Fig. 2a). As described above, V-ATPase is a large multi-subunit complex composed of a cytosolic ATP-hydrolyzing V_1_ domain and a membrane-embedded V_o_ proton channel.^8^ Of the eight V-ATPase subunits identified as putative STAT3-associating proteins, one was a V_o_ subunit (ATP6V0D1) and seven were V_1_ subunits, and ATP6V1A was the strongest candidate based on the amount of protein co-immunoprecipitating with STAT3-Flag, the number of peptides identified, and repeated identification in independent experiments (Fig. 2a). Similar association of endogenous STAT3 with the V-ATPase complex was demonstrated by its ability to co-precipitate with endogenous ATP6V1A as analyzed by immunoblotting and nano-LC-MS/MS (Fig. 2b; Supplementary information, Fig. S2a and b), and to co-precipitate with the V-ATPase complex purified with hemagglutinin-tagged ATP6V1A (Fig. 2c). Super resolution-structured illumination microscopy (SR-SIM) confirmed the close proximity of endogenous STAT3 and ATP6V0D1 in intact HeLa cells (Fig. 2d, e). Likewise, proximity ligation assay (PLA), which detects protein colocalization with single-molecule resolution, gave strong, punctate PLA signals for STAT3 and ATPV6A1 in HeLa cells, as well as in non-transformed pancreatic duct epithelial cells (H6C7) and mammary fibroblasts (HMF3), but not in their STAT3-depleted counterparts (Fig. 2f, g). Notably, PLA for STAT3 and other abundant lysosomal membrane proteins LAMP1 and CD63 gave practically no signal (Fig. 2h).

**Fig. 3:**
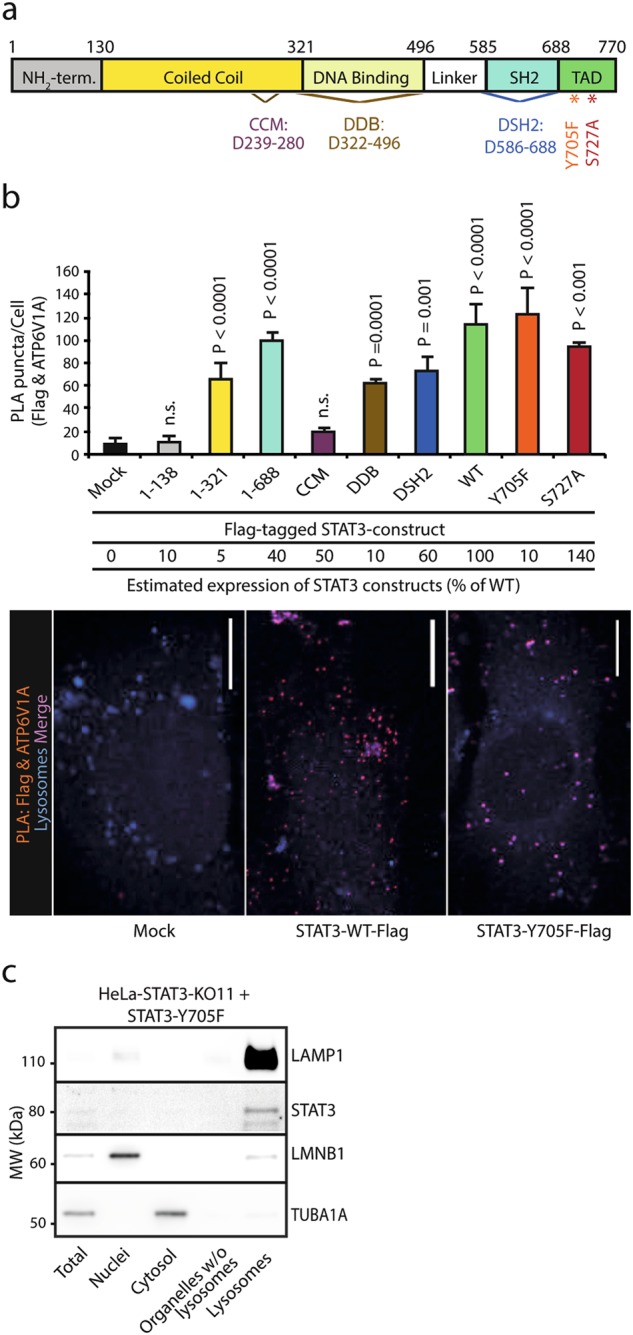
STAT3 interacts with V-ATPase via its coiled-coil domain. **a** Domain structure of STAT3 with mutations used in **b** indicated below. SH2, Src homology 2 domain; TAD, transactivation domain. **b** Quantification of PLA (anti-Flag and anti-ATP6V1A) puncta in HeLa cells expressing the indicated Flag-tagged STAT3 constructs (top). Error bars, SD of three independent experiments with ≥ 10 cells analyzed/sample. *P*-values were calculated by one-way ANOVA combined with Dunnett’s multiple comparisons test. Rough estimates of the relative expression levels of Flag-tagged STAT3 constructs are shown below the histogram as percentages of the expression of the wild-type STAT3. See Supplementary information, Fig. S3b for representative immunoblots. Bottom, representative images of PLAs taken with 60×  magnification using Zeiss LSM700 confocal microscope. The optimal slice thickness (∼350 nm) was defined by the Zeiss zen software. Lysosomes were visualized with cascade blue dextran. Scale bar, 10 µm. **c** Representative immunoblots of the indicated proteins in total cell lysates or the indicated fractions of HeLa-STAT3-KO cells reconstituted with STAT3-Y705F (20 µg protein/lane). *n* = 3

To characterize the association between STAT3 and the V-ATPase complex, we performed mutagenesis of STAT3 and investigated the ability of the Flag-tagged STAT3 mutants to associate with the V-ATPase complex by PLA employing antibodies against Flag and ATP6V1A in HeLa cells. The first 138 amino acids of the NH_2_ terminus of STAT3 failed to colocalize with ATP6V1A, whereas NH_2_-terminal STAT3 fragments of 321 or 688 amino acids showed roughly similar colocalization to the wild-type protein (Fig. 3a, b; Supplementary information, Fig. S3a). Thus, the coiled-coil domain residing between the amino acids 138 and 321 appeared to be essential for the interaction. To corroborate this hypothesis, we deleted a highly conserved sequence between residues 239 and 280 of the STAT3 coiled-coil domain (Supplementary information, Fig. S3a). The obtained coiled-coil mutant (CCM) failed to associate with ATP6V1A in spite of its relatively high expression in HeLa cells (Fig. 3b and Supplementary information, Fig. S3b). On the contrary, mutants lacking the entire DNA-binding domain (ΔDB) or SH2 domain (ΔSH2), as well as single amino acid mutants defective in mitochondrial (Ser727Ala; S727A) or transcriptional (Tyr705Phe; Y705F) functions of STAT3 colocalized with ATPV1A approximately as well as or even better than the wild-type protein (Fig. 3b). Notably, cells expressing STAT3-Y705F-Flag mutant showed a similar PLA signal to cells expressing the wild-type STAT3-Flag in spite of ∼10-fold higher expression level of the latter (Fig. 3b and Supplementary information, Fig. S3b). Correspondingly, immunoblot analyses of subcellular fractions of HeLa cells expressing STAT3-Y705F-Flag revealed almost exclusively lysosomal localization of this mutant (Fig. 3c). These data strongly suggest that the coiled-coil domain of STAT3 associates with the V-ATPase complex, and that this association does not require phosphorylation of Tyr705 or Ser727 residues.

### STAT3 enhances lysosomal acidification and activity

**Fig. 4:**
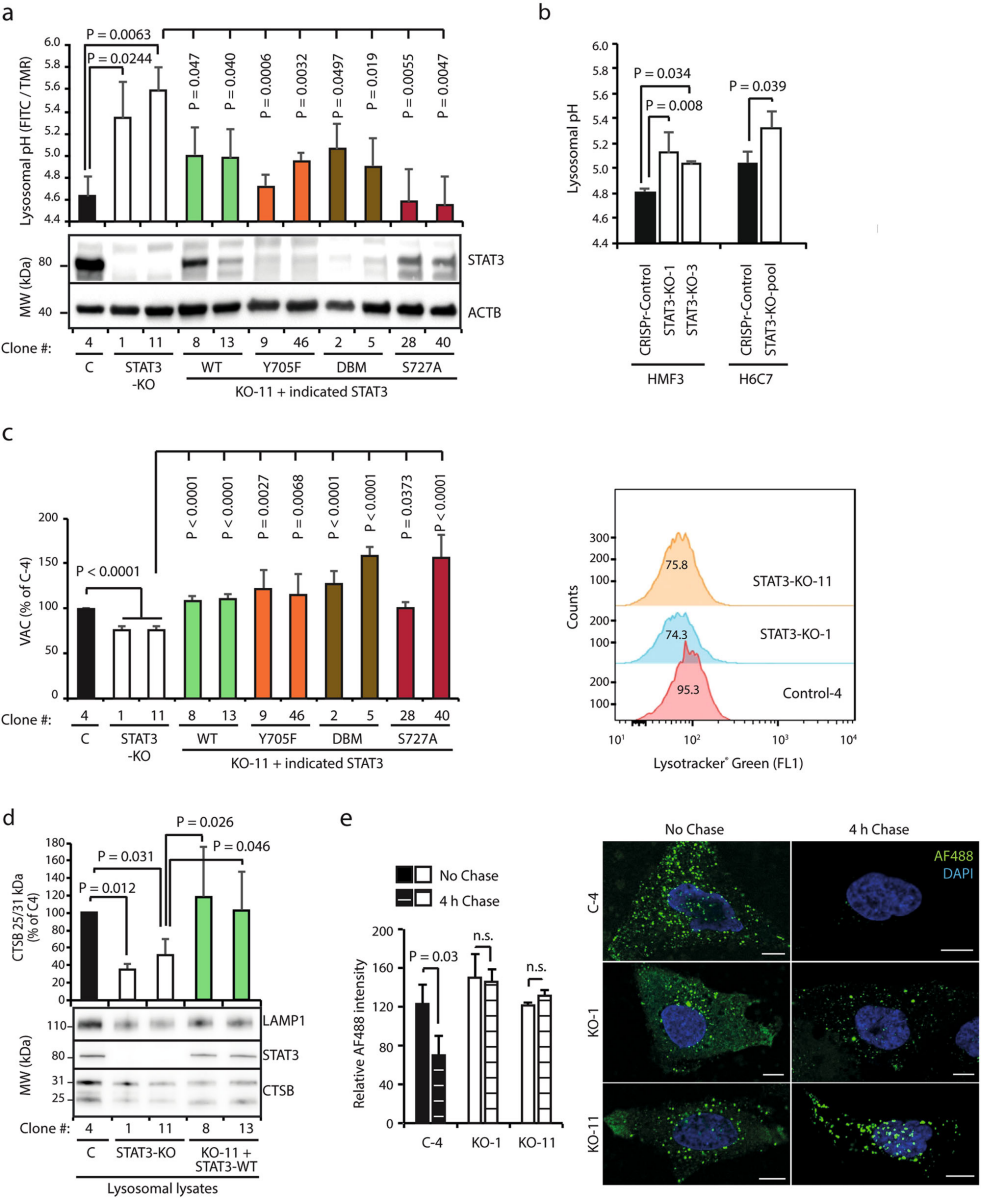
STAT3 regulates lysosomal pH and activity. **a** Lysosomal pH determined by FITC/TMR ratio in a HeLa CRISPR control cell clone (C-4), STAT3-KO clones (KO-1 and −11), and KO-11 clone reconstituted with wild-type (WT) or mutated (Y705F, DBM, S727A) STAT3 constructs. Representative immunoblots show STAT3 and ACTB (loading control) protein levels in the clones. Standard curve for pH measurements is shown in Supplementary information, Fig. S4a. **b** Lysosomal pH determined as in **a** in CRISPR control and STAT3-KO HMF3 and H6C7 cells. Standard curve for pH measurements is shown in Supplementary information, Fig. S4a. **c** Volume of acidic compartment (VAC) in HeLa cell clones described in **a** analyzed by flow cytometer after 5 min staining with 75 nM Lysotracker Green. Relative fluorescence intensities are shown on the left. A representative flow cytometry profile is shown on the right. For other flow cytometry profiles and gating of the cells, see Supplementary information, Fig. S4c and d. **d** Representative immunoblots of LAMP1, STAT3, and cathepsin B (CTSB) in lysosomal lysates of the indicated HeLa cell clones. The histogram shows ratios between the active (25 kDa) and inactive (31 kDa) CTSB as percentages of the value in C4 control clone. **e** AlexaFluor 488-Dextran degradation in the indicated HeLa clones loaded with 0.4 mg/ml AlexaFluor 488-dextran for 20 min, washed, and fixed with or without a 4 h chase period. Representative images taken with 60× magnification using Zeiss LSM700 confocal microscope are shown on the right. Error bars, SD of ≥ 3 independent experiments. A minimum of 10 cells/sample were analyzed in **a**, **b**, and **e**. *P*-values were calculated by one-way ANOVA combined with Dunnett’s multiple comparisons test (**a**, **c**) or two-stage linear step-up procedure of Benjamini, Krieger, and Yekutieli (**b**, **d**) for multiple comparisons, or by two-tailed, homoscedastic Student’s *t*-test (**e**)

Prompted by the association of STAT3 with the V-ATPase complex, we investigated whether STAT3 regulated the activity of the V-ATPase. For this purpose, we depleted *STAT3* from HeLa cells using CRISPR-Cas9-mediated gene editing and estimated lysosomal pH based on the fluorescence intensity ratio of pH-sensitive fluorescein (FITC) and pH-insensitive tetramethylrodamine (TMR) in cells loaded with dextran coupled to these dyes.^36^ The estimated lysosomal pH values in STAT3-deficient HeLa clones were increased from the 4.6 in control cells to 5.3 and 5.6 in STAT3-KO-1 and STAT3-KO-11 clones, respectively (Fig. 4a and Supplementary information, Fig. S4a). In non-transformed HMF3 mammary fibroblasts and H6C7 pancreatic duct epithelial cells, CRISPR-Cas9-mediated STAT3 depletion increased the lysosomal pH significantly, albeit to a lesser extent than in HeLa cells (Fig. 4b and Supplementary information, Fig. S4a). The increased lysosomal pH in HeLa-STAT3-KO-11 clone was partially reverted in stable clones reconstituted with wild-type STAT3 (Fig. 4a). Reconstitution with Y705F, DNA-binding defective and S727A mutants of STAT3 resulted in a similar or even better rescue than wild-type STAT3 in spite of their lower expression levels (Fig. 4a). As we did not obtain stable clones expressing STAT3-CCM, the mutant that failed to colocalize with the V-ATPase as analyzed by PLA (Fig. 3b), we investigated its ability to rescue the lysosomal pH phenotype of HeLa-STAT3-KO-11 cells after transient transfection. Contrary to the wild-type STAT3, which significantly reduced the lysosomal FITC/TMR ratio following a transient transfection, STAT3-CCM failed to do so (Supplementary information, Fig. S4b). To validate the role of STAT3 in the acidification of the lysosomal compartment, we measured the volume of the acidic compartment of HeLa cells loaded with Lysotracker Green by flow cytometry. In line with increased lysosomal pH observed by FITC/TMR ratio measurements in HeLa-STAT3-KO clones, the STAT3-depleted cells had a significantly lower capacity to accumulate Lysotracker Green than the control cells, and this phenotype was effectively rescued by the reconstitution with the wild-type STAT3 or its Y705F, S727A, and DNA-binding defective mutants (Fig. 4c; Supplementary information, Fig. S4c and d). Further supporting the role of STAT3 in the acidification of lysosomes, HeLa-STAT3-KO cells had reduced maturation of cathepsin B (CTSB) and longer half-life of lysosomal dextran conjugated to pH-insensitive AlexaFluor 488 (Fig. 4d, e).

### STAT3 stimulates V-ATPase-mediated hydrolysis of ATP

**Fig. 5:**
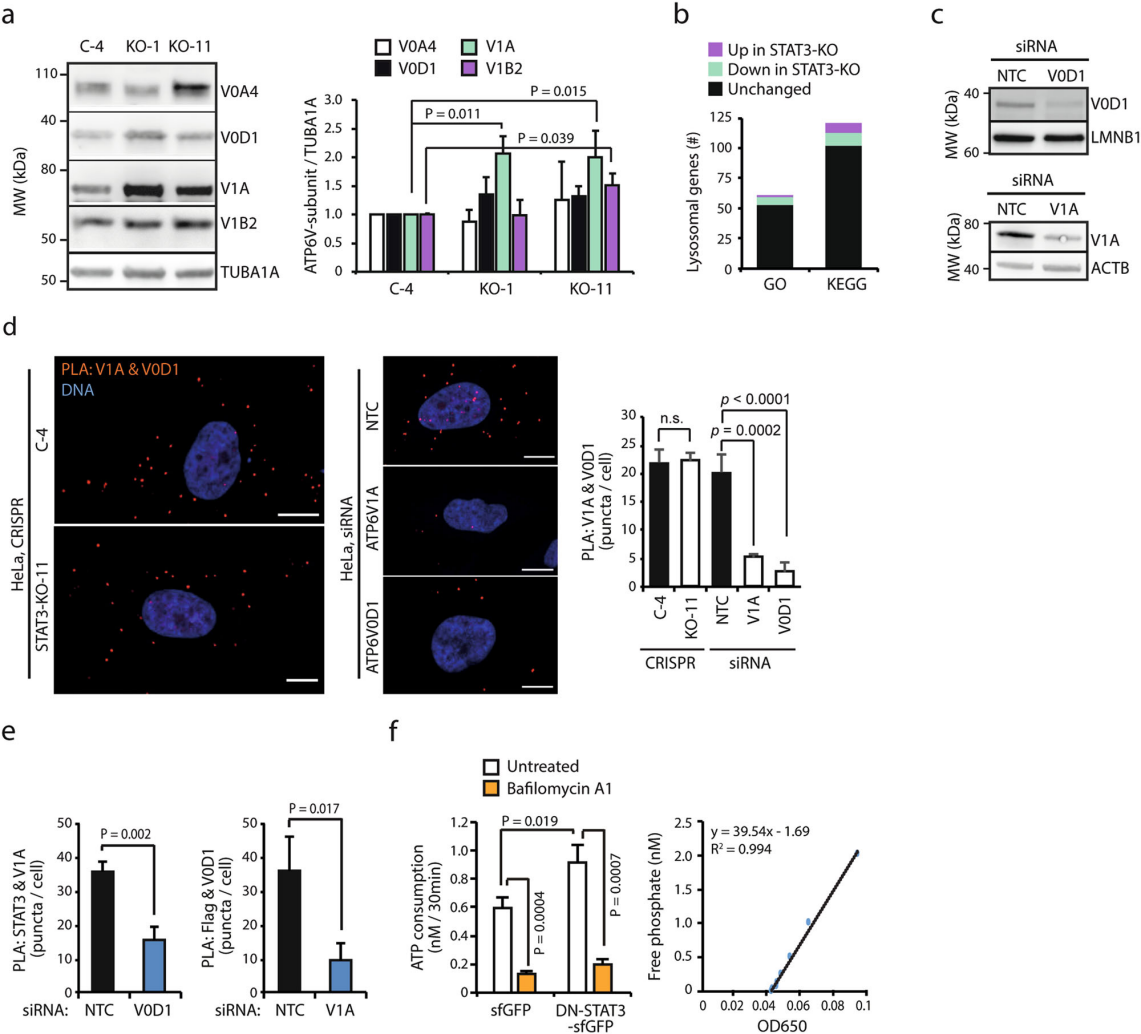
STAT3 enhances V-ATPase activity. **a** Representative immunoblots (left) and quantification (right) of the indicated V-ATPase subunits from lysates of HeLa CRISPR control clone (C-4) and STAT3-KO clones (KO-1 and −11). TUBA1A served as a loading control. **b** Numbers of lysosomal genes whose expression analyzed by RNA-Seq was decreased or increased over ≥ 1.5-fold (*P* ≤ 0.05) in HeLa-STAT3-KO cells as compared to HeLa-C4 control cells. Lysosomal genes were defined as genes whose protein products localize to lysosomes according to either Gene Ontology or Kyoto Encyclopedia of Genes and Genomes databases. See Supplementary information, Fig. S5b for the list of altered genes. **c** Representative immunoblots of the indicted proteins from lysates of HeLa cells transfected with the indicated siRNAs 72 h earlier. *n* = 3. **d** Representative images (left) and quantification (right) of PLA puncta with antibodies against ATP6V1A (V1A) and ATP6V0D1 (V0D1) in HeLa CRISPR control (C-4) and STAT3-KO (STAT3-KO-11) cells, as well as in HeLa cells transfected with the indicated siRNAs 72 h earlier. DNA was stained with DAPI. Images were taken with 60× magnification using Zeiss LSM700 confocal microscope. The optimal slice thickness (∼350 nm) was defined by the Zeiss zen software. Scale bar, 10 µm. **e** Quantification of PLA puncta with antibodies against STAT3 and V1A in HeLa cells (left) or Flag and V0D1 in HeLa-STAT3-Flag cells (right). Cells were transfected with the indicated siRNAs 72 h earlier. **f** Activity of V-ATPase in the presence of 30 µg/mL superfolder-GFP (sfGFP; control) or ΔN-STAT3-sfGFP. V-ATPase was immunoprecipitated with anti-HA magnetic beads from lysosomal lysates of HeLa cells transiently transfected with pCDNA3.1-HA-ATP6V1A. When indicated, the samples were treated with 100 nM bafilomycin A1. Right, standard curve for the measurement of the free phosphate ion used to estimate the ATP consumption. Protein blot for recombinant proteins is shown in Supplementary information, Fig. S5c. Error bars, SD of ≥ 3 independent experiments. A minimum of ten cells/sample were analyzed in **d**, **e**. *P*-values were calculated by one-way ANOVA combined with Dunnett’s multiple comparisons test (**a**, **d**), DEseq2 (**b**), or by two-tailed, homoscedastic Student’s *t*-test (**e**, **f**)

The ability of Y705F and DNA -binding defective mutants of STAT3 to effectively rescue the lysosomal pH phenotype in STAT3-KO cells strongly suggested that STAT3 acidifies lysosomes independently of its transcriptional activity. To substantiate this hypothesis, we sequenced the transcriptomes of HeLa-C4 and HeLa-STAT3-KO cells and compared the expression levels of mRNAs for lysosomal proteins (Supplementary information, Fig. S5a and b). STAT3 depletion did not reduce the expression of mRNAs for major V-ATPase subunits (Supplementary information, Fig. S5a). On the contrary, several of them and their corresponding proteins (e.g., ATPV1A, ATPV1B2, and ATP6V0D1) were upregulated in STAT3-KO cells (Fig. 5a and Supplementary information, Fig. S5a). Out of all the genes whose protein products according to Gene Ontology (www.geneontology.org/page/go-enrichment-analysis) or Kyoto Encyclopedia of Genes and Genomes (www.genome.jp/kegg/pathway.html) datasets localize to lysosomes, only 13 and 10 genes had over 1.5-fold reduced and increased expression, respectively, in HeLa-STAT3-KO cells as compared with control cells (Fig. 5b and Supplementary information, Fig. S5b). To our knowledge, none of the altered genes, except for the upregulated V-ATPase subunits, have been reported to regulate lysosomal pH. These data suggest that STAT3 stimulates lysosomal acidification by a posttranscriptional mechanism. Thus, we tested whether STAT3 regulated the assembly of V_1_ and V_o_ domains of the V-ATPase complex by investigating their proximity by PLA employing antibodies against ATPV1A and ATP6V0D1. The PLA was validated by the efficient reduction in the number of PLA puncta upon small interfering RNA (siRNA)-mediated depletion of either ATPV1A or ATP6V0D1 (Fig. 5c, d). HeLa-STAT3-KO-11 cells had as many PLA puncta as the control cells, indicating that STAT3 does not control the assembly of the V-ATPase complex (Fig. 5d). In line with this, the significant reduction in the colocalization of STAT3 with ATP6V1A and ATP6V0D1 following the siRNA-mediated depletion of ATP6V0D1 and ATP6V1A, respectively, suggested that STAT3 associates with the assembled V-ATPase complex (Fig. 5c, e). Finally, the ability of STAT3 to directly enhance V-ATPase activity was supported by *in vitro* reconstitution experiments showing that recombinant STAT3 enhanced the bafilomycin A1-sensitive ATPase activity of the immunopurified V-ATPase complex (Fig. 5f). Taken together, these data suggest that STAT3 enhances lysosomal acidification by stimulating the ATPase activity of the V_1_ domain in the assembled V-ATPase complex on the lysosomal membrane.

### Cytosolic pH regulates STAT3 localization and STAT3 localization regulates cytosolic pH

**Fig. 6:**
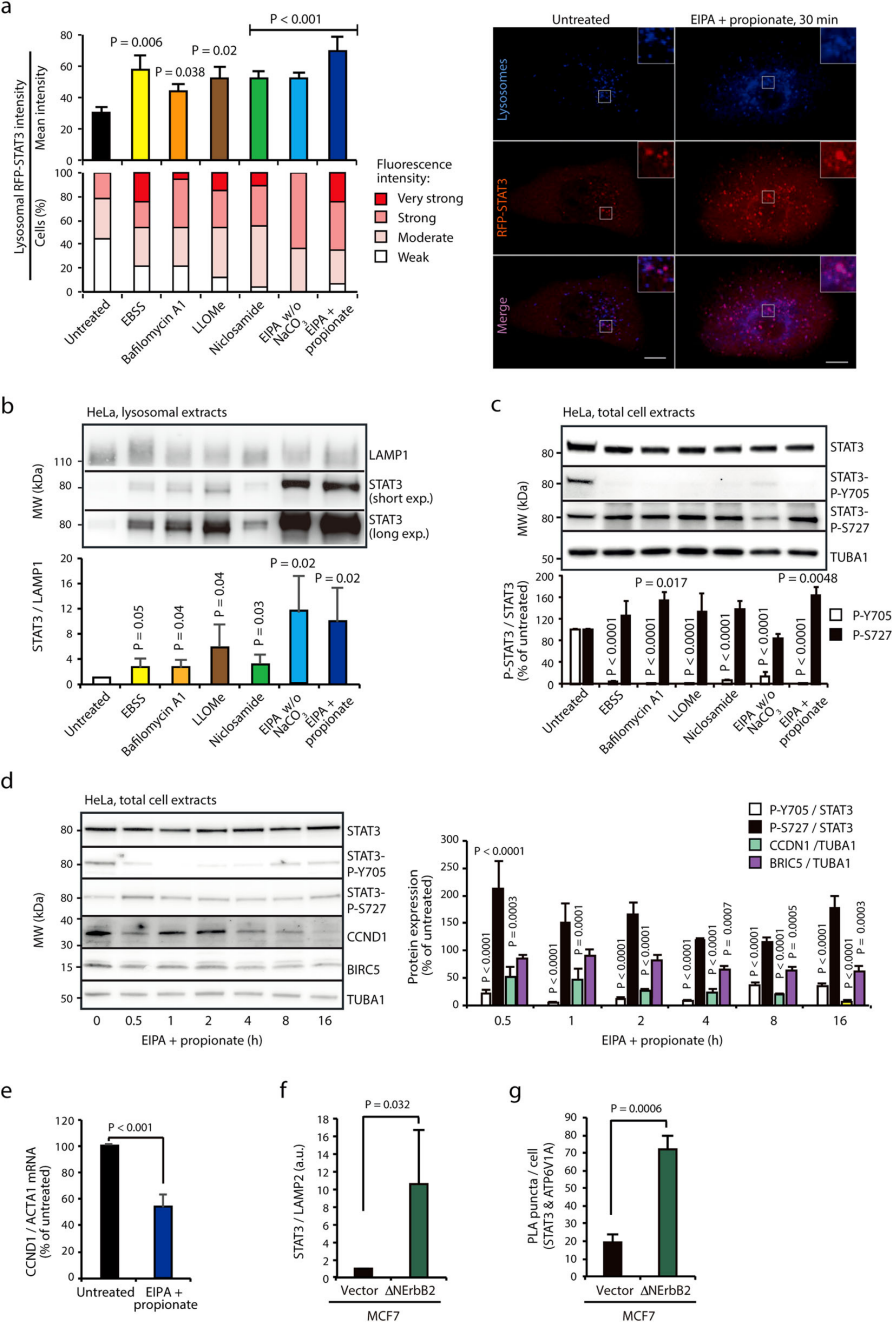
STAT3 regulates cytosolic pH. **a** Intensity of lysosomal RFP-STAT3 in A549-RFP-STAT3 cells loaded with 0.4 mg/ml cascade blue dextran for 1 h, chased for 5 h, and treated with EBSS for 4 h, 0.1 µM bafilomycin A1 or 10 µM niclosamide for 1 h, 25 µM EIPA in the absence of NaHCO_3_ or the presence of 50 mM propionate for 0.5 h, or with 1 mM LLOMe for 15 min. Histograms show mean lysosomal RFP intensities/cell (top) and distribution of lysosomal RFP intensities in a cell population (bottom). Representative images of live cells taken with 60× magnification using Zeiss LSM700 confocal microscope are shown on the right. Scale bar, 10 µm. **b** Representative immunoblots of LAMP1 and STAT3 in lysosomal lysates of HeLa cells left untreated or treated as in **a**, except for LLOMe treatment that was for 1 h. The histogram shows relative ratios of STAT3/LAMP1. Cytosolic acidification caused by these treatments is shown in Supplementary information, Fig. S6a. **c** Representative immunoblots of the indicated proteins in lysates of HeLa cells treated as in **b**. The histogram shows relative ratios of P-Y705-STAT3 and P-S727-STAT3. **d** Representative immunoblots of the indicated proteins in lysates of HeLa cells treated with 25 µM EIPA + 50 mM propionate for the indicated times. CCND1, cyclin D1; BIRC5, survivin. *n* = 3. **e** Quantitative PCR analysis of *CCND1* mRNA levels in HeLa cells left untreated or treated with 25 µM EIPA + 50 mM propionate for 4 h. *ACTA1* mRNA served as an internal control. **f** Quantification of STAT3/LAMP2 ratios in immunoblots of proteins from lysosomes immunopurified with anti-LAMP1. See Supplementary information, Fig. S6d for a representative blot. **g** Quantification of PLA (anti-STAT3 and anti-ATP6V1A) puncta in MCF7-vector and MCF7-p95DNErbB2 cells. Error bars, SD of ≥ 3 independent experiments. *P*-values were calculated by one-way ANOVA combined with Dunnett’s multiple comparisons test (**b**, **c**, **d**, **f** and **g**) or by two-tailed, homoscedastic Student’s *t*-test (**e**) in comparison with the untreated cells

Inspired by the ability of STAT3 to enhance the V-ATPase-mediated transfer of protons from the cytosol to lysosomes, we tested whether its subcellular localization could be regulated by changes in intracellular pH. To disrupt the intracellular pH equilibrium, we starved cells for amino acids to enhance the V-ATPase activity,^37^ or exposed them to five short (15–60 min) treatments that acidify the cytosol (Supplementary information, Fig. S6a), i.e., (i) V-ATPase inhibitor bafilomycin A1,^38^ (ii) lysosomal membrane destabilizer l-leucyl-l-leucine methyl ester (LLOMe),^39^ (iii) niclosamide, a protonophore that carries protons across membranes,^40^ or (iv) ethylisopropyl amiloride (EIPA), an inhibitor of plasma membrane NHE1, in the absence of NaHCO_3_/CO_2_ buffer system or (v) in the presence of propionic acid (propionate).^41^ All tested treatments significantly enhanced the association of RFP-STAT3 with lysosomes in A549-RFP-STAT3 cells as demonstrated by increased RFP-STAT3 puncta formation and significantly enhanced lysosomal fluorescence intensity of RFP-STAT3 (Fig. 6a). In HeLa cells, all tested treatments triggered a significant increase in lysosomal STAT3 as demonstrated by significantly increased STAT3/LAMP1 ratios in immunoblots of lysosomal lysates (Fig. 6b). These increases were likely due to the translocation of STAT3 from other cellular compartments, as none of the short treatments induced detectable changes in the expression levels of STAT3 or LAMP1 (Fig. 6b, c). In addition, all cytosol-acidifying treatments effectively reduced the phosphorylation of Tyr705-STAT3 in HeLa and A549 cells (Fig. 6c, d; Supplementary information, Fig. S6b). In agreement with the dependence of the transcriptional activity of STAT3 on P-Tyr705, cytosolic acidification reduced the levels of proteins encoded by known STAT3 target genes, *CCND1* and *BIRC5*, as analyzed by immunoblotting (Fig. 6d), *CCND1* mRNA as analyzed by quantitative PCR (qPCR) (Fig. 6e), and mRNA levels of *CCND1*, *MYC*, and 179 other predicted STAT3 target genes as analyzed by whole transcriptome sequencing (Supplementary information, Fig. S6c). Thus, acidification of the cytosol triggers a rapid dephosphorylation of P-Tyr705-STAT3 accompanied by translocation of STAT3 to the lysosomes, where it can promote the neutralization of the acidic cytosol by enhancing the proton pumping activity of the V-ATPase complex.

Most cancers depend on aerobic glycolysis to generate ATP, and several oncogenes enhance this process by upregulating the expression of glucose transporters and glycolytic enzymes.^42^ This results in increased cytosolic lactate production. Thus, we tested whether oncogene-mediated activation of glycolysis and subsequent acid production are associated with enhanced translocation of STAT3 to the lysosomes. For this purpose, we used MCF7 cells with inducible expression of N-terminally truncated active form of ErbB2 oncogene, p95ΔNErbB2,^43^ which enhances glycolysis by upregulating lactate dehydrogenase expression.^44,45^ Immunopurified lysosomes from MCF7-p95ΔNErbB2 cells had over ten times higher STAT3/LAMP1 ratio than control MCF7 cells, whereas the level of total STAT3 was similar (Fig. 6f and Supplementary information, Fig. S6d). Increased lysosomal localization of STAT3 in MCF7-p95ΔNErbB2 cells was confirmed by PLA for STAT3 and ATP6V1A (Fig. 6g). Taken together, these data suggest that the increased acid production in cancer cells increases the amount of lysosomal STAT3, which in turn enhances the proton pumping activity of the V-ATPase thereby possibly assisting in the maintenance of alkaline cytosol in spite of increased acid load.

### STAT3 protects cells against cytosolic acidification

**Fig. 7:**
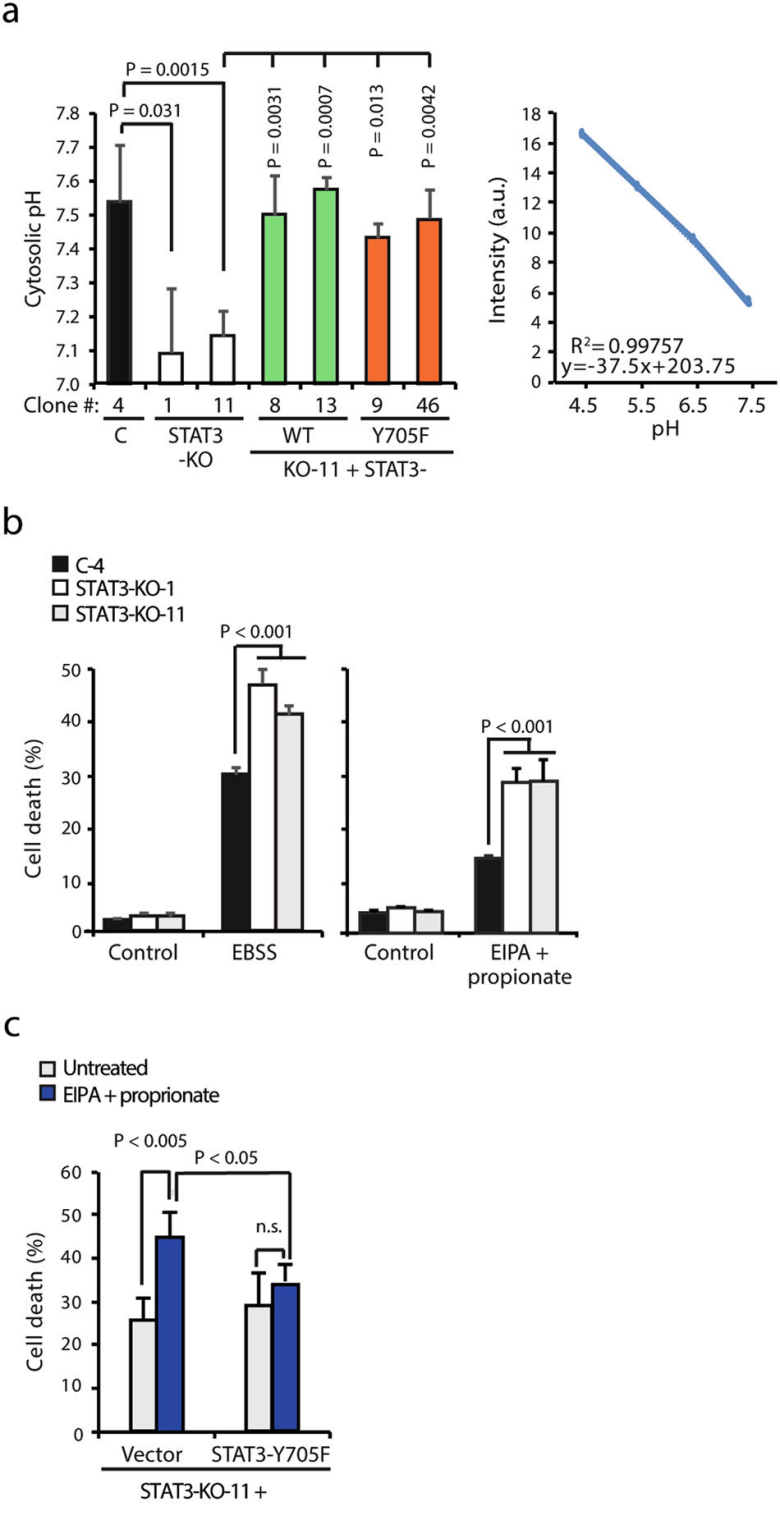
Lysosomal STAT3 protects cells against cytosolic acidification. **a** Cytosolic pH in the indicated HeLa clones determined by image analysis of cells loaded with pHrodo™ Green AM. Standard curve is displayed on the right. **b**, **c** Cell death of the indicated HeLa cell clones (**b**) and HeLa-STAT3-KO-11 cells transiently transfected with vector or STAT3-Y705F (**c**) left untreated, starved in EBSS for 6 h, or treated with 25 µM EIPA + 50 mM propionate for 24 h was determined by propidium iodine uptake. Error bars, SD of ≥ 3 independent experiments. *P*-values were calculated by one-way ANOVA combined with Dunnett’s multiple comparisons test (**a**, **b**) or by two-tailed, homoscedastic Student’s *t*-test (**c**)

As mentioned above, malignant transformation and cancer progression depend on reverse pH gradient, and cancer cells maintain higher cytosolic pH (pH_c_) than non-transformed cells.^3^ Consistent with the role for STAT3 in the maintenance of alkaline pH_c_ of cancer cells, the pH_c_ of both STAT3-KO clones was significantly lower (7.09 and 7.14) than the pH_c_ of 7.54 in control HeLa cells (Fig. 7a). The decreased pH in STAT3-KO cells was effectively reverted by reconstitution with either the wild-type STAT3 or its mainly lysosomal and transcriptionally defective Y705F mutant (Fig. 7a). Moreover, both STAT3-KO clones died significantly more than control cells when exposed to stresses affecting intracellular proton equilibrium, i.e., amino acid starvation or cytosolic acidification (Fig. 7b). Notably, the sensitivity to cytosolic acidification could be effectively rescued by the ectopic expression of either the wild-type STAT3 or its Y705F mutant (Figs. 3c and 7c). Thus, in response to cytosolic acidification, P-Tyr705-STAT3 is dephosphorylated and more STAT3 is recruited to lysosomal membranes to enhance the V-ATPase activity and cell survival, whereas the transcriptional activity of STAT3 is inhibited.

## DISCUSSION

In addition to its canonical role as a nuclear transcription factor, STAT3 is today recognized as a multifunctional protein that has non-canonical roles both in nucleus and cytoplasm. The data presented here reveal a previously unrecognized role for the cytoplasmic STAT3 in the control of intracellular pH homeostasis via its association with the lysosomal V-ATPase.

Mitochondrial STAT3 has received ample attention in recent years,^32^ whereas the putative functions of STAT3 in other cytoplasmic compartments have been largely ignored. Using multiple state-of-the art methods, we demonstrate here a dynamic colocalization of STAT3 with the lysosomal compartment. Live-cell imaging of endogenous STAT3 fused to fluorescent proteins and immunocytochemistry employing anti-STAT3 antibodies revealed predominantly punctate cytoplasmic STAT3 signals that colocalized almost exclusively with lysosomal markers in A549, SKOV3 and HeLa cells. Furthermore, STAT3 co-precipitated with meticulously purified lysosomal fractions of HeLa cells that were devoid of detectable amounts of protein markers for other cellular compartments. Contrary to the predominantly lysosomal localization of STAT3 puncta observed by imaging, only ∼5% of the total STAT3 co-purified with the lysosomes of cells growing in normal culture conditions. This discrepancy could be due to a high local concentration of STAT3 in individual lysosomes resulting in intensive local fluorescence or dissociation of the loosely attached STAT3 from the lysosomes during the organelle purification.

Punctate cytoplasmic STAT3 staining pattern has been previously reported in cells treated with cytokines or growth factors. During receptor-mediated endocytosis, Tyr705-phospshorylated STAT3 colocalizes with endocytic vesicles in transit from the cell membrane to the perinuclear region.^46–48^ In pulmonary arterial hypertension, this centripetal trafficking of STAT3 is disturbed in pulmonary arterial cells, where STAT3 and P-Tyr705-STAT3 are sequestered inside the lysosomes.^49^ In our experiments employing untreated cancer cells, STAT3 did not colocalize with early endosomes, and the majority of lysosomal STAT3 was not phosphorylated at Tyr705 residue. Furthermore, and contrary to the lysosomal localization of STAT3 induced by pulmonary arterial hypertension, STAT3 resided on the cytosolic side of the lysosomal membrane in normally growing cancer cells. Therefore, this lysosomal pool of STAT3 appears to be distinct from the pool of STAT3 previously detected in the endo-lysosomal compartment. It should be noted that the lysosomal localization of STAT3 was not limited to the three cancer cell lines mentioned above, but was also observed in non-transformed human mammary fibroblasts and pancreatic duct epithelial cells. Therefore, it is surprising that except for two proteomics datasets that list STAT3 as a lysosomal protein,^50,51^ the apparently widespread association of STAT3 with the lysosomal outer membrane has not been reported earlier. This may be due to its very loose and dynamic association with the lysosomes that is easily disturbed even by mild detergents commonly used to permabilize cells for immunocytochemistry or immunoprecipitation. In line with this, the dynamic lysosomal localizations of several signaling complexes, e.g., the intensively studied mammalian target of rapamycin complex 1 and AMP-activated protein complex, were discovered relatively late, while having been missed by most studies intending to reveal the lysosomal proteome.^52–54^

Further supporting the existence of STAT3 on lysosomal membranes, we identified an association between the V-ATPase complex and an evolutionarily highly conserved sequence in the coiled-coil domain of STAT3. In repeated experiments, both endogenous and exogenous STAT3 co-precipitated with various V-ATPase subunits, and the catalytic ATP6V1A subunit of the cytoplasmic V_1_ domain was the most abundant and most frequently identified binding partner. As the V_1_ complex does not form in the absence of ATP6V1A subunit and since the individual subunits of the V_1_ and V_o_ complex are poorly soluble when not in complex with the other subunits (K.M., unpublished data), we were not able to verify the direct association between STAT3 and ATP6V1A in vitro. The significant reduction of the PLA signal from ATPV0D1 and STAT3 upon depletion of the ATP6V1A subunit supported, however, the idea that STAT3 associated with V-ATPase via the ATP-hydrolyzing V_1_ complex. This was further substantiated by the ability of STAT3 to enhance the ATPase activity of the V-ATPase complex in vitro. The characterization of the molecular details of this association will require extensive structural analyses of the entire membrane-embedded V-ATPase complex, which are out of the scope of this study.

The V-ATPase controls lysosomal luminal pH in tightly regulated manner that promptly responds to extracellular and intracellular cues.^9^ This requires constant and precise control of the speed of proton pumping, which is achieved by fine-tuning the activity and density of the V-ATPase. The molecular mechanisms underlying this regulation are only beginning to emerge. The proton pumping activity of the V-ATPase is rapidly and effectively controlled by the assembly status of the V_1_ and V_o_ domains,^55^ and in a slower fashion by the relative expression levels of the V-ATPase subunits,^56^ whereas the density is determined mainly by the expression levels of the subunits and various membrane fusion and fission events.^57^ Here, we introduce STAT3 as a potent regulator of intracellular pH, whose depletion results in significant increase in lysosomal pH and decrease in cytosolic pH, respectively. We propose that STAT3 enhances the ATPase activity of the cytosolic V_1_ complex via a direct association with the complex or a regulatory protein tightly associated with it. This hypothesis is based on the ability of recombinant STAT3 to promote the bafilomycin A1-sensitive ATPase activity of the V-ATPase complex in a reconstitution assay in vitro, as well as by meticulous exclusion of alternative possibilities. It is highly unlikely that the STAT3-mediated intracellular pH regulation is mediated by its canonical or non-canonical transcription-regulating functions. First, the pH phenotype of STAT3-depleted cells was effectively rescued not only by the wild-type STAT3 but also by STAT3 mutants (Y705F, ΔSH2, and DNA-binding mutants) unable to activate the transcription of classical STAT3 target genes. Second, STAT3 depletion reduced neither the protein expression of major V-ATPase subunits nor mRNA levels of any genes encoding V-ATPase subunits or other proteins reported to regulate lysosomal pH, e.g., lysosomal members of the chloride channel or transient receptor potential channel families or known regulators of the V-ATPase assembly. Third, a recent proteomics study on the STAT3-mediated regulation of lysosomal composition did not identify STAT3 dependancy for any known pH regulating lysosomal proteins.^58^ Finally, genes encoding such proteins were not identified as non-canonical transcriptional targets of STAT3, which can be activated in the absence of Tyr705 phosphorylation.^59^ As STAT3 depletion failed to reduce the assembly of V_1_ and V_o_ domains as demonstrated by unaltered proximity of ATP6V1A and ATP6V0D1 in STAT3-depleted cells, it is also unlikely that STAT3-mediated regulation of intracellular pH involves the association of the two domains. In light of these data, the most probable explanation is that STAT3 directly regulates the activity of the V-ATPase. Interestingly, a change in the luminal pH of the lysosomes has been suggested to induce a change in the V-ATPase conformation, which facilitates its binding to Arf6 nucleotide-exchange factor thereby regulating endosome recycling.^60^ Thus, it is tempting to speculate that similar pH-regulated conformational changes in either the V-ATPase or STAT3 could regulate the association of STAT3 and the V-ATPase complex to assist in the reestablishment of the intracellular pH homeostasis. The putative relevance of such local STAT3-mediated regulation of the V-ATPase activity is supported by our data showing that the neutralization of the lysosomal lumen and/or acidification of the cytosol can increase the recruitment of STAT3 to the lysosomal membrane up to tenfold in only 30 min. In this manner, STAT3 can rapidly respond to disturbances of intracellular pH equilibrium by enhancing the rate of proton exclusion from the cytosol to the lysosomal lumen and thereby enhance the survival of cells upon increased acid load. In line with this, STAT3 depletion sensitized cells to cell death induced by cytosolic acidification and this phenotype was effectively reverted by wild-type STAT3 as well as its Y705F mutant. Even though the lysosomal compartment may have a limited capacity to receive protons from the considerably larger volume of the cytosol, this process combined with removal of lysosomal protons to the extracellular space via lysosomal exocytosis could be an effective way of retaining alkaline cytosol even during long-lasting acidifying stress caused, e.g., by increased glycolysis in cancer cells. Hence, STAT3 may enhance cancer cell survival by augmenting the adaptation of the cells to the acidic tumor environment and increased acid production.

In conclusion, we have identified an intriguing feedback loop between cytosolic pH and STAT3 that contributes to the maintenance of alkaline pH_c_, acidification of lysosomes and transcriptional activity of STAT3 in cancer cells (Supplementary information, Fig. S7). In spite of the critical role of STAT3 in human malignancies and decades of intensive research aiming at the development of drugs interfering with STAT3 activity, there are no clinically approved drugs directly targeting STAT3 so far. One possible explanation is that most efforts have focused on the inhibition of the STAT3’s nuclear function, which may indirectly result in an increase in its extranuclear activities, i.e., regulation of mitochondrial electron transfer chain as suggested earlier,^30,31^ and maintenance of alkaline pH_c_ and lysosomal function as proposed here. The identification of these non-transcriptional roles of STAT3 will hopefully inspire further studies that can result in more holistic strategies to target STAT3 in cancer treatment.

## MATERIALS AND METHODS

### Reagents and resources

Sources of reagents and resources used are listed in Supplementary information, Table S1.

### Cell culture

Human A549 non-small cell lung carcinoma (male), HeLa cervix carcinoma, and SKOV3 ovarian carcinoma were obtained from American Type Culture Collection (ATCC). The cells were authenticated by ATCC and used within 6 months after thawing. Human pancreatic duct epithelial cells (H6C7) were purchased from Kerafast. Human immortalized mammary fibroblasts (HMF3) were kindly provided by Michael J. O’Hare (Ludwig Institute for Cancer Research, London, UK).^61^ Lenti-X^TM^ 293T human embryonic kidney epithelial cells transformed with Adenovirus type 5 DNA were from Clontech (632180). A549-triple cells (here referred to as A549-RFP-STAT3 cells) were kindly provided by Dmitry Malkov (Sigma-Aldrich, St. Louis, Missouri, USA).^34^ Human MCF7 mammary carcinoma cells stably transfected with inducible pTRE-p95ErbB2 or corresponding pTRE vector were established and cultured as described previously.^13,43^ The expression of p95ErbB2 was induced by washing off the tetracycline (1 µg/ml) three passages before the experiment. H6C7 cells were cultured in serum free media (SFM). Other cells were cultured in Dulbecco’s modified Eagle’s medium (DMEM) supplemented with 10% heat-inactivated fetal calf serum and penicillin/streptomycin (complete medium), and for H6C7 cells also with 2 mM glutamine. Cells were maintained at 37 °C and 5% CO_2_. All cell lines were found negative for mycoplasma using Venor^®^GeM Classic PCR kit.

### STAT3 mutagenesis

To perform site-directed mutagenesis of STAT3, pCDNA3.1-DYK-STAT3 was PCR amplified using KOD Xtreme™ Hot Start DNA Polymerase and primers listed in Supplementary information, Table S2. Larger deletions (ΔDB and ΔSH2) were performed as described previously,^62^ using primers listed in Supplementary information, Table S2. Amplified DNA digested with *Dpn*I was amplified in *Escherichia coli* DH5α. The obtained sequence-verified plasmids and EF.STAT3-Y705F.Ubc.GFP^63^ were used as templates for the construction of STAT3 expression plasmids described in Supplementary information, Table S3.

### Transfections

If not otherwise stated, transfections were performed using TurboFectin 8.0 or Lipofectamine 3000 transfection agents according to the manufacturer’s instructions.

siRNAs were transfected with Lipofectamine RNAmax at 20 nM according to the manufacturer’s protocol and cells were analyzed 72 h later.

To create stable cell lines, lentiviral and packaging plasmids were transfected into subconfluent Lenti-X^TM^ 293T cells. After 48 h, virus particles were collected with PEG-it™ Virus Precipitation Solution according to the manufacturer’s instructions. HeLa cells growing on six-well plates (2 × 10^5^ cells/well) were treated with 5 µl of the obtained virus suspension supplemented with 8 µg/mL protamine sulfate and spin-infected by centrifugation at 2,400× *g* for 90 min. After 48 h, cells were transferred to 10 cm petri dish and the medium was supplemented with 200 µg/mL hygromycin B. After ~3 weeks of selection, stable clones were picked and analyzed by immunoblotting to identify successfully transfected clones.

For transposon-mediated transfection, expression plasmids and super transposase vector (1:1) were co-transfected into HeLa cells growing on six-well plates (2 × 10^5^ cells/well). After 48 h, cells were transferred into a 10 cm petri dish and the medium was supplemented with 1 µg/mL puromycin. After ~2 weeks of selection, stable clones were picked and validated by immunoblotting.

### CRISPR/Cas9-mediated gene editing

STAT3-knockout cells were generated using CRISPR/Cas9-mediated gene editing essentially as described previously.^64^ Single-guide RNA targeting STAT3 was created by cloning appropriate primers into pxpr001^65^ and transfected to HeLa, H6C7, or HMF3 cells. After 2 weeks of selection in medium containing 1 µg/mL puromycin, clones were picked up and validated by immunoblotting and sequencing.

The EGFP-STAT3 tagging CRISPR gRNA was generated by cloning the guide sequences Cr-Uni and Cr-STAT3 into the pMA-SpCas9-g1 and pMA-SpCas9-g2 gRNA expression vectors, respectively.^66^ The pAAV-EGFP-MMEJ vector was generated by amplifying the EGFP coding sequences from ptfLC3 (kindly provided by Dr. Yoshimori^67^) with specific primers listed in Supplementary information, Table S3. The PCR product was digested by *Not*I and ligated into a pAAV-MCS vector. All plasmids were further validated by Sanger sequencing.

To generate the SKOV3-EGFP-STAT3 cells, SKOV3 cells growing on six-well plates (2.5 × 10^5^ cells/well) were transfected with 400 ng pAAV-EGFP-MMEJ, 100 ng pMA-STAT3-gRNA, 100 ng pMA-MMEJ-gRNA, 300 ng pSpCas9(BB)-2A-Puro (PX459^68^) and 100 ng pNeDaKo-Neo employing XtremeGENE^TM^ 9 DNA transfection reagent according to the manufacturer’s instructions. Cells were passaged to a 10 cm petri dish 24 h later and after an additional 24 h, the medium was supplemented with 0.7 µg/mL G418 for 10 days. G418-resistant cells were further expanded and enriched for EGFP-positive cells by fluorescence-activated cell sorting (FACS core facility, Aarhus University). SKOV3-EGFP-STAT3 cells derived from a single-cell clone were identified and validated by Sanger sequencing and immunostaining.

### Labeling of lysosomes and other organelles

Organelles were visualized 2–3 days after transfection with the indicated plasmids encoding appropriate organelle markers fused to BFP, i.e., pLAMP1-BFP or pLAMP2-BFP for lysosomes, mTagBFP2-MannII-N-10 for Golgi,^69^ mTagBFP2-Rab5a-7 for early endosomes,^69^ mito-BFP for mitochondria,^65^ and BFP-KDEL for endoplasmatic reticulum.^65^ Alternatively, lysosomes were visualized by loading the cells with 0.4 mg/mL cascade blue dextran (10 kDa) or 0.25 mg/mL AlexaFluor 594-dextran (10 kDa) for 1 h in 37 °C cell incubator, followed by washing with Dulbecco′s phosphate-buffered saline (DPBS) and 5 h incubation in 37 °C cell incubator. The samples were then analyzed either live or after 20 min fixation in 4% paraformaldehyde (PFA) with Zeiss LSM700 confocal laser scanning microscope with 60× objective.

### Subcellular fractionation and lysosome purification

Subconfluent cells grown in 15 cm petri dishes in 20 mL complete medium were treated for 24 h with 2 mL iron-dextran solution (53 mg/mL in deionized water; prepared essentially as described previously^70^), washed in DPBS, and cultured for additional 24 h in complete medium. Then, cells were scraped off, washed in DPBS, and ruptured on ice in SCA buffer (20 mM Hepes-KOH, pH 7.5, 10 mM KCl, 1.5 mM MgCl_2_, 1 mM EDTA, 1 mM EGTA, 250 mM sucrose) with freshly added 1× protease inhibitor cocktail using a Dounce glass homogenizer (BioVision, 1998-1) until ~90% of cells were permeable for trypan blue. Ruptured cells were centrifuged at 1,000× *g* for 10 min at 4 °C. Nuclear pellets were lysed in TBS lysis buffer (50 mM Tris-Cl, pH 7.5, 150 mM NaCl, 0.5% CHAPS, 1% octyl-β-glucoside) with 1× protease inhibitor cocktail and the supernatant containing cytosol and organelles was loaded to LS MACS Separation Columns on octoMACS^TM^ Separator magnet stand. First, 1 mL of eluate was collected, columns were washed twice with 0.5 mL SCA buffer, and lysosomes were eluted with 1 mL SCA buffer after removing the columns from the magnet. Both eluates were centrifuged at 20,000× *g* for 20 min, supernatant from the first eluate (cytosol) was collected and pellets (organelles without lysosomes and lysosomes) were lysed in TBS lysis buffer with 1× protease inhibitor cocktail.

Alternatively, lysosomes were immunopurified with anti-LAMP1 antibodies. Subconfluent cells growing on petri dishes were scraped in ice-cold DMEM, washed three times with cold phosphate-buffered saline (PBS), and resuspended in SCA-low EDTA buffer (250 mM sucrose, 20 mM Hepes, 10 mM KCl, 1.5 mM MgCl_2_, 0.1 mM EDTA, 1 mM EGTA, pH 7.5) with freshly added protease and phosphatase inhibitor cocktail. Cell suspension (6 × 10^7^ cells in 800 µL) was homogenized in gentleMACS using program “tissue: h_mito_tissue_01” and centrifuged at 750× *g* for 5 min at 4 °C. The supernatant (light membrane fraction) was collected and rotated with 0.225 µg primary antibody at 4 °C for 15 min before adding 10 µL MicroBeads coupled to goat anti-rabbit antibody for 1 h. The suspension (3× 150 µL) followed by 500 µL SCA-low EDTA buffer was applied to MS Columns equilibrated with SCA-low EDTA buffer supplemented with 25 U/mL benzonase in the magnetic field of the octoMACS^TM^ Separator. After removing the column from the magnetic stand, lysosomes were eluted with 3× 100 µL SCA-low EDTA buffer.

### Immunodetection

#### Immunoblotting

Cells were lysed in Laemmli sample buffer (125 mM Tris, pH 6.7, 20% glycerol, 140 mM SDS) supplemented with complete protease inhibitor cocktail. After addition of 0.05 M dithiothreitol and bromophenol blue, boiling and separation by 4%–20% gradient SDS-polyacrylamide gel electrophoresis, proteins were transferred onto polyvinylidene difluoride membranes using Bio-Rad Trans-Blot Turbo system. Membranes were blocked with PBS containing 5% milk and 0.1% Tween-20, and stained with the indicated primary antibodies and appropriate peroxidase-conjugated secondary antibodies listed in Supplementary information, Table S1. CTSB antibody was kindly provided by Dr Ekkehard Weber (Martin Luther University Halle-Wittenberg, Halle, Germany).^71^ The signal was detected with Clarity Western ECL Substrate and Luminescent Image Reader, and quantified by densitometry with Image Studio Lite software.

#### Immunocytochemistry

Cells grown on glass coverslips were fixed with 4% PFA or ice-cold methanol, quenched with 50 mM ammonium chloride in DPBS, permeabilized and blocked in 5% goat serum, 1% bovine srum albumin (BSA) in DPBS supplemented with 0.3% Triton X-100 or 0.1% saponin, and stained with the indicated primary and secondary antibodies listed above. Coverslips were mounted with Prolong Gold Antifade mounting medium with DAPI. Images were acquired using a Zeiss LSM700 microscope with Plan-Apochromat 63×/1.40 Oil DIC M27 objective and Zen 2010 software (all equipment and software from Carl Zeiss, Jena, Germany). Pinholes were set so that the section thickness was equal for all channels and ≤ 1 AU. Cell contours (*n* > 20) were defined manually and green and red thresholds were set up in single channel mode and retained for all samples in an experiment. SR-SIM was performed using a 63×, 1.4 numerical aperture, oil-immersion objective lens, and an sCMOS PCO.edge camera mounted on an Elyra PS.1 microscope. Samples were illuminated with 488 and 561 nm lasers passed through diffraction gratings of 34 and 42 µm, respectively. Processing was performed with Zen software black edition 2012 and the channels were aligned in *x*, *y*, *z* according to a matrix calculated with an image of 100 nm beads recorded in the same conditions as the sample. Three-dimensional analyses were done by Fuji software and remodeling was done by Zen 2012 black software.

#### Immunoprecipitation

Cells washed with PBS and scraped off in SCA buffer supplemented with 1× protease inhibitor cocktail, were ruptured using a Dounce glass homogenizer until ~90% of cells were permeable for trypan blue. The supernatant obtained after 10 min centrifugation at 1,000× *g* at 4 °C was further centrifuged at 20,000× *g* for 20 min at 4 °C and the pellet (organelles) was lysed in TBS lysis buffer supplemented with 1× protein phosphatase and protease inhibitor cocktails on ice. The obtained lysate (150 µg protein) was precleared by 1 h incubation with 10 µL control IgG conjugated with magnetic beads and the supernatant aspirated on magnetic stand was rotated for 2 h with 20 µL specific antibodies (rabbit anti-HA or mouse anti-FLAG) or appropriate control IgG conjugated to magnetic beads at 4 °C. Magnet-purified beads were washed three times with TBS lysis buffer, once with TBS with 300 mM NaCl at 4 °C, and proteins were eluted in 25 µL Laemmli lysis buffer for 5 min at 95 °C before immunoblot analysis.

#### Proximity ligation assay

PLA assay was performed with Duolink In Situ Red Starter Kit according to the manufacturer’s instructions. Briefly, cells grown on coverslips were fixed with 4% PFA for 20 min, permeabilized in buffer 1 (PBS with 1% BSA and 0.3% Triton X-100) for 10 min and blocked in Duolink blocking buffer. Samples were then incubated for 18 h at 4 °C with the indicated primary antibodies diluted in buffer 1 supplemented with 5% goat serum, washed 3× 5 min in buffer 1, and incubated with Duolink secondary antibodies for 1 h at 37 °C. After washing 2× 5 min in Duolink washing buffer A, samples were incubated for 30 min at 37 °C with 1:40 dilution of Duolink ligase in Duolink ligation buffer and washed 2× 2 min in Duolink washing buffer A. Finally, samples were incubated for 100 min at 37 °C with 1:80 dilution of Duolink polymerase in Duolink amplification buffer, washed for 2× 10 min in 1× Duolink washing buffer B and for 1 min in 0.01× washing buffer B, and mounted with ProLong™ Gold Antifade Mountant. Images were taken by LSM700 with 60× magnification and analyzed by open source Image J software.

### In vivo crosslinking

In vivo crosslinking was performed by incubating cells in 3 mL fresh culture media containing 2.5 mM dithiobis(succinimidyl propionate) (DSP) for 10 min at 37 °C and 5% CO_2_. DSP was quenched by adding 330 µL of 1 M Tris-HCl (pH 8.0) and incubating for another 10 min. After two washes with cold PBS, cells were lysed in 500 µl TBS lysis buffer supplemented with 1× protease and phosphatase inhibitor cocktails on ice for 20 min.

### Protein identification by mass spectrometry

Anti-FLAG immunoprecipitation from lysates of lysosomal extracts from HeLa cells transfected with pBCMV-STAT3-Flag-puro or empty vector 3 days earlier, and anti-STAT3 and mIgG2a immunoprecipitation from total lysates of HeLa cells after in vivo crosslinking were performed according to the protocol described above. Interacting proteins were identified and quantified by nano-LC-MS/MS, essentially as previously described.^72^ Briefly, each gel lane was cut into 1× 1 mm pieces and cysteine residues were blocked by reduction and alkylation using tris(2-carboxyethyl)phosphine and iodoacetamide, respectively. In-gel digestion was performed using trypsin and resulting peptides were extracted from gel pieces using acetonitrile and trifluoroacetic acid, and finally purified on PepClean C-18 Spin columns. LC-MS/MS was performed on an EASY nanoLC coupled to a Q Exactive Plus Hybrid Quadrupole-Orbitrap Mass Spectrometer. Peptide samples were separated on a PepMap C-18 reverse phase column (25 cm length, 75 µm inner diameter, and 2 μm particle size) and eluted by a 90 min linear gradient of acetonitrile (4%–40%) containing 0.1% formic acid. The MS was operated in a data-dependent mode, automatically switching between MS and MS2 acquisition, with mass resolution of 70,000 and 17,500, respectively. Up to ten most intense ions were fragmented per every full MS scan by higher-energy collisional dissociation. Dynamic exclusion of 10 s was applied and ions with single charge or unassigned charge states were excluded from fragmentation. MaxQuant software version 1.5.2.8 was applied for protein identification and label-free quantification by means of peptide peak areas.^73^ The MS raw files were searched against a database consisting of 20,197 *Homo sapiens* protein sequences downloaded from UniProt.^74^ Carbamidomethylation of cysteines was set as fixed modification, whereas methionine oxidation and protein N-terminal acetylation were set as dynamic modifications. The false discovery rate (FDR) was assessed by searching against a reverse decoy database^75^ and FDR thresholds of protein and peptide identification were both set to 0.01.

### pH measurements

Lysosomal pH was estimated based on the fluorescence intensity ratio of pH-sensitive FITC and pH-insensitive TMR essentially as described previously.^36^ In brief, cells were loaded with 2.5 mg/mL 70 kDa dextran coupled to FITC and TMR in complete medium for 18 h, washed with DPBS, and incubated for additional 5 h with complete medium without dextran. Images of dextran-loaded cells were acquired by LSM700 confocal laser scanning microscope (Zeiss) and the FITC/TMR ratio was calculated by open source Image J software. Standard curves used to estimate lysosomal pH were created by similar analysis of cells incubated with a series of Hepes buffers (145 mM KCl, 10 mM glucose, 1 mM MgCl_2_, 10 µM nigericin, 20 mM Hepes) with pH ranging from 4.5 to 7.0. After transient transfections, pH changes were expressed as arbitrary units based on FITC/TMR ratio, the lower ratio indicating higher pH.

The relative volume of the acidic compartment of trypsinized cells stained for 5 min with 75 nM Lysotracker Green DND-26 and washed in PBS was estimated by flow cytometry analysis employing BD FACSVerse™ instrument. Data were collected with BD FACSuite v1.0.6 and analyzed by Flowjo V10 software.

To estimate the cytosolic pH, cells washed with Live Cell Imaging Solution were incubated for 30 min in 37 °C in the same solution containing 1:1,000 dilution of pHrodo™ Green AM Intracellular pH Indicator and 1:100 dilution of PowerLoad™ concentrate, washed with Live Cell Imaging Solution, and analyzed by LSM700 confocal laser scanning microscope. Standard curves used to estimate cytosolic pH were created by similar analysis of cells incubated with a series of pH calibration buffers (pH 4.5, 5.5, 6.5, and 7.5) supplemented with 10 µM valinomycin and 10 µM nigericin (Intracellular pH Calibration Kit).

### STAT3 recombinant protein generation

To produce recombinant N-terminally truncated and C-terminally superfolder-GFP-tagged STAT3 (ΔN-STAT3-sfGFP), the sequence coding for amino acid residues 127–770 of STAT3 was PCR amplified with primers listed in Supplementary information, Table S2 and ligated into the cloning site of the pETM11SUMO3sfGFP vector. The obtained pETM11SUMO3ΔNSTAT3sfGFP plasmid was used to transform competent *E. coli* BL21 Star^TM^(DE3) cells. After 18 h shaking at 16 °C, cells were lysed in the lysis buffer (50 mM Tris-HCl, pH 7.5, 500 mM NaCl, 20 mM imidazole, 0.5 mM dithiothreitol (DTT)) supplemented with 1× complete EDTA-free protease inhibitor cocktail, 1× BugBuster Protein Extraction Reagent, and 50 units/mL Benzonase Nuclease, and centrifuged at 21,000× *g* for 45 min at 4 °C. The supernatant was filtered (0.2 µm) and passed through Ni-NTA affinity resins (Qiagen, 30210), which were washed in lysis buffer before elution of N-terminally hexahistidine- and SUMO3 domain-tagged recombinant ΔN-STAT3sfGFP protein in Tris buffer (50 mM Tris-HCl, pH 7.5, 250 mM NaCl) supplemented with 300 mM imidazole. The protein was dialyzed against Tris buffer overnight at 4 °C in the presence of 200:1 (mol:mol) of in house-purified SenP2 protease to cleave off the N-terminal tags. Finally, the protein was loaded on the Superdex 200 size-exclusion chromatography column (GE Healthcare), and the fractions containing the ΔN-STAT3sfGFP were pooled and stored at −80 °C. sfGFP control protein was prepared in a similar manner using pETM11SUMO3sfGFP vector for transformation.

### V-ATPase assay

To purify V-ATPase, two 15 cm petri dishes with subconfluent HeLa cells were transfected with pCDNA3.1HA-ATP6V1A plasmid (5 µg/plate). Three days later, cells were scraped off in SCA buffer supplemented with 1× protease inhibitor cocktail and ruptured using a Dounce glass homogenizer until ∼90% of cells were permeable for trypan blue. Ruptured cells were centrifuged at 1,000× *g* for 10 min and the obtained supernatant was further centrifuged at 20,000× *g* for 20 min. The pellet was lysed in 450 µL TBS lysis buffer supplemented with 1× protease inhibitor cocktail, incubated on ice for 30 min, and centrifuged at 20,000× *g* for 1 min. After determining the protein concentration, the supernatant containing 150 µg protein was precleared by rotation with 12 µL magnetic bead-conjugated rabbit IgG for 1 h at 4 °C. The precleared supernatant aspirated on magnetic stand was then rotated with 20 µL magnetic bead-conjugated rabbit mAb against HA for 2 h at 4 °C and the supernatant was aspirated on magnetic stand. The beads were washed 3× 5 min in 500 µL of TBS lysis buffer and suspended in 50 µL of V-ATPase reaction buffer (40 mM HEPES, pH 7.5, 1 mM MgCl_2_, 100 mM KCl, 0.25 mM Sucrose, 5 µg/mL Oligomycin, 1 mM DTT) supplemented with 1× protease inhibitor cocktail.

To measure the V-ATPase activity, 15 µL anti-HA beads (V-ATPase) were mixed with 4.5 µg ΔN-STAT3sfGFP or 4.5 µg sfGFP (negative control) and the volume was adjusted to 150 µL with V-ATPase reaction buffer. The reaction was started by adding 1 mM ATP (final concentration) and incubating the samples for 30 min at 30 °C. The samples and free phosphate standard dilutions were then transferred to 96-well plates (50 µL/well) and the reactions were stopped by adding 100 μL BIOMOL Green Reagent. Plates were incubated at 25 °C for 20–30 min before determining OD620nm in a Varioskan Flash Multimode Reader. The amount of ATP hydrolyzed was calculated using the standard curve created by the free phosphate dilutions analyzed on the same plate.

### Dextran degradation assay

Subconfluent cells were loaded with 0.4 mg/mL AlexaFluor 488-dextran (10 kDa) for 20 min and washed twice with PBS. The samples for the analysis of AlexaFluor 488-dextran uptake were then fixed with 4% PFA for 20 min, whereas the parallel samples for the analysis of AlexaFluor 488-dextran degradation were incubated in complete medium for additional 4 h before fixation. Images were acquired using a Zeiss LSM700 microscope with EC Plan-Apochromat 63×/1.40 Oil DIC M27 objective. Fluorescence intensities were determined by open source Image J software.

### RNA sequencing

The mRNA and non-coding RNAs were enriched by removing rRNA with RNaseH. Target RNAs were fragmented into short fragments in the fragmentation buffer and cDNAs were synthesized using the RNA fragments as templates for N6 random primer, followed by end reparation and ligation to adapters. The quantity and quality of the cDNA libraries were assessed using an Agilent 2100 BioAnalyzer. Finally, the libraries were sequenced on the BGISEQ-500 with 50 single-end read.

Sequencing reads that contained adapters, had low quality, or aligned to rRNA were filtered off before mapping. Clean reads were aligned to the hg19 UCSC RefSeq (RNA sequences, GRCh37) using bowtie2. Fragments per kilobase of transcript per million mapped reads values were obtained by transforming mapped transcript reads using RSEM. Differential expression analysis was performed by DESeq2. Differentially expressed genes were defined as genes with fold change ≥ 1.5 and *P*-value ≤ 0.05. Clean reads were mapped to the hg19 genome using hisat2.

### Quantitative PCR

qPCR was performed using SuperScript™ III Platinum™ SYBR™ Green One-Step qPCR Kit according to the manufacturer’s instructions.

### Identification of STAT3 target genes

Putative STAT3 target genes were defined by annotating the 2001 common peaks from the STAT3 ChipSeq datasets GSM935276_hg19_wgEncodeSydhTfbsHelas3Stat3IggrabPk.narrowPeak.gz and GSM935591_hg19_wgEncodeSydhTfbsMcf10aesStat3Etoh01StdPk.narrowPeak.gz from HeLa-S3 and MCF10A-Er-Src cells, respectively, to promoter sequences 2 kb upstream and 1 kb downstream of transcription start sites.

### Cell death assay

Cells (5 × 10^3^) plated on 96-well plates were treated as indicated, and stained with 3 µg/mL propidium iodide and 5 µg/mL Hoechst 33342 for 1 min. Percentage of dead cells (propidium iodide positive/Hoechst positive) of all cells (Hoechst positive) was quantified by Celigo Cell Imaging Cytometer.

### Quantification and statistical analysis

All experiments were performed a minimum of three times and results are expressed as mean ± SD from ≥ 3 independent experiments. For all image-based analyses, ≥ 10 randomly chosen cells/sample were analyzed. Data obtained from the lysotracker staining, PLA, lysosomal pH measurements, and cell death assays comparing multiple genetically modified clones were analyzed using one-way ANOVA with multiple comparison by Dunnett’s multiple comparisons test. Cathepsin maturation examined by western blotting and lysosomal pH measurement in HMF3 cells were analyzed using one-way ANOVA with multiple comparison by two-stage linear step-up procedure of Benjamini, Krieger, and Yekutieli. Significance of differences in mRNA expression levels analyzed by RNA sequencing (RNA-Seq) were defined by DEseq2. Other data were analyzed using by two-tailed, homoscedastic Student’s *t*-test.

### Data resources

The MS proteomics data have been deposited to the ProteomeXchange Consortium (www.ebi.ac.uk/pride) via the PRIDE^76^ partner repository with the dataset identifier PXD006788. GEO accession number for RNA-Seq data is GSE108495. Other datasets generated during the current study will be available from the corresponding author upon reasonable request.

## Electronic supplementary material

Supplementary information, Figure S1

Supplementary information, Figure S2

Supplementary information, Figure S3

Supplementary information, Figure S4

Supplementary information, Figure S5

Supplementary information, Figure S6

Supplementary information, Figure S7

Supplementary information, Table S1

Supplementary information, Table S2

Supplementary information, Table S3
