# Supplementary material for: STAT3 associates with vacuolar H+-ATPase and regulates cytosolic and lysosomal pH
Source: Cell Res. 2018 Aug 20;28(10):996–1012. doi: 10.1038/s41422-018-0080-0 (PMC6170402; doi:10.1038/s41422-018-0080-0)
Supplement: Supplementary file 1 — Supplementary information, Figure S1 [file 41422_2018_80_MOESM1_ESM.pdf]

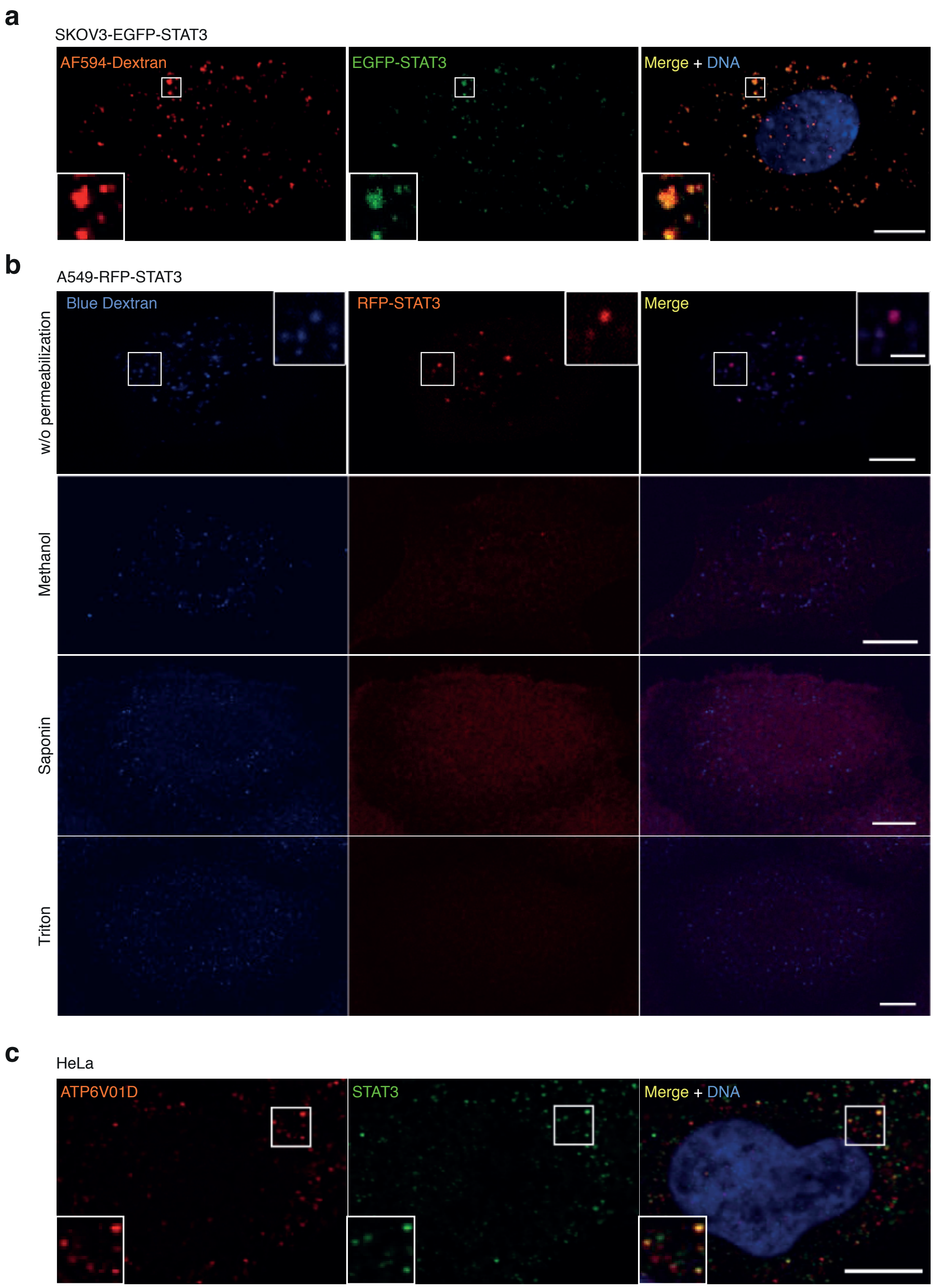

Supplementary Figure S1. STAT3 co-localizes with lysosomes
a Representative images of live SKOV3-EGFP-STAT3 cells loaded for 1 h with 0.25 mg/ml AlexaFluor®594 and chased for 5 h. Cells were counter-stained with Hoechst before the analysis.
b Representative images of A549-RFP-STAT3 cells loaded with 0.4 mg/ml cascade blue-dextran (lysosomes) for 1 h and chased for 5 h. Samples were fixed with 4% paraformaldehyde without permeabilization or permeabilized with methanol for 5 min at -20° C, or 0.1% saponin or 0.3% triton-X100 for 10 min at 20° C.
c Representative images of HeLa cells stained with anti-ATP6V01D, anti-STAT3 and DAPI. Samples were fixed with 4% paraformaldehyde and permeabilized with 0.1% saponin for 10 min at 20° C.

All images were taken with 60x magnification using Zeiss LSM700 confocal microscope.The marked areas (white squares) are magnified in lower left or upper right corners. Scale bars, 10 μm in main figures and 2 μm in magnifications (**b**).All experiments were performed at least 3 times with similar results.
