# Supplementary material for: STAT3 associates with vacuolar H+-ATPase and regulates cytosolic and lysosomal pH
Source: Cell Res. 2018 Aug 20;28(10):996–1012. doi: 10.1038/s41422-018-0080-0 (PMC6170402; doi:10.1038/s41422-018-0080-0)
Supplement: Supplementary file 2 — Supplementary information, Figure S2 [file 41422_2018_80_MOESM2_ESM.pdf]

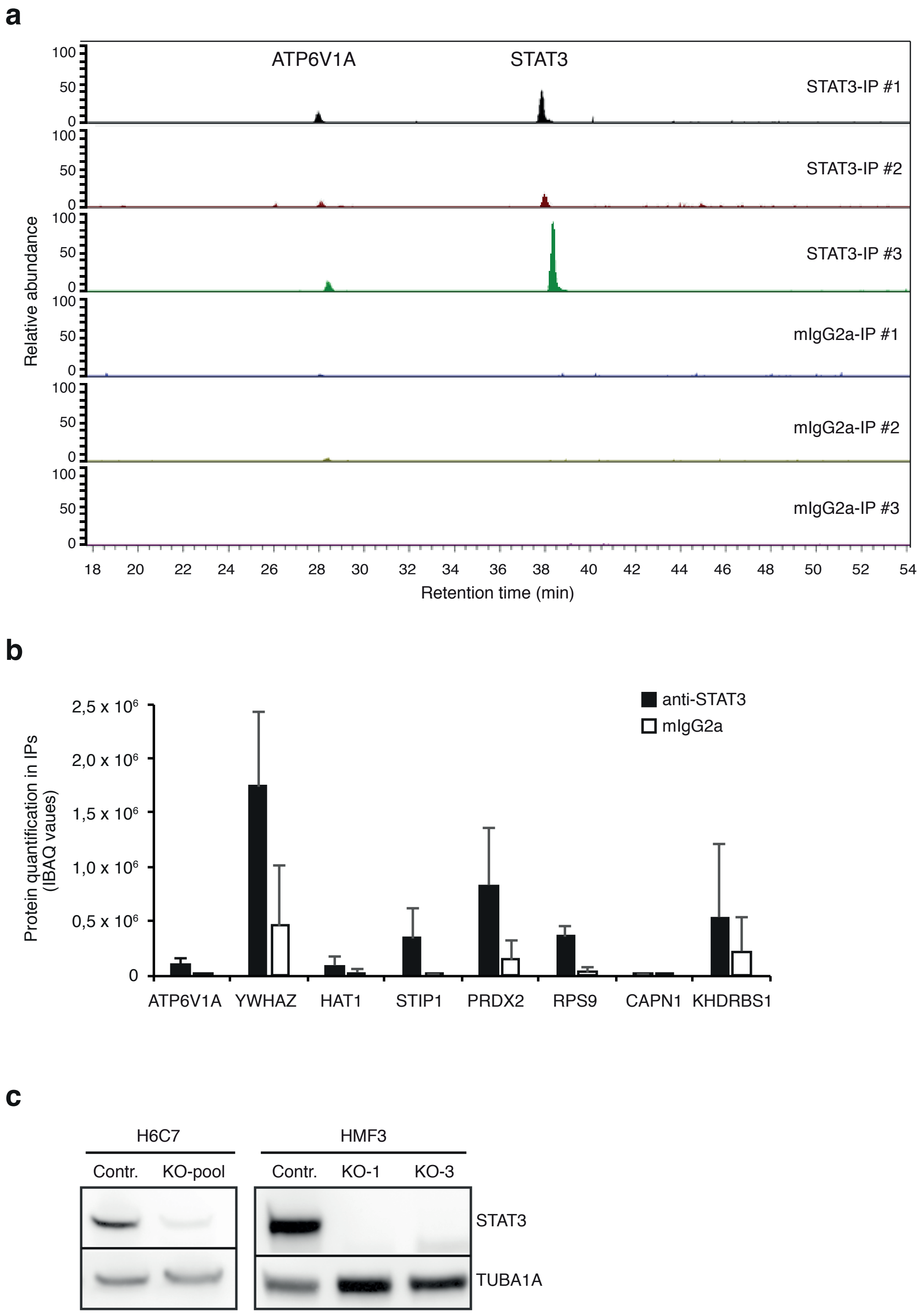

Supplementary Figure S2. STAT3 interacts with the V-ATPase
a Quantitation of STAT3 and ATP6V1A in three independent samples immunoprecipitated with mouse IgG2a antibody against STAT3 or control mouse IgG2a from total lysates of HeLa cells. The peaks are extracted ion chromatograms (XIC) of exact mass (< 5 parts per million) peptide signal of STAT3 (LLGPGVNYSGCQITWAK) at 38 min and ATP6V1A (PANHPLLTGQR) at 28 min. The selected STAT3 and ATP6V1A peptides are unique for the representative protein, and have in the samples been identified by MS/MS fragmentation, on Q Exactive Plus MS, with P values of 1.3 * 10-18 and 0.00011, respectively.
b Label-free quantitative values (iBAQ values from Maxquant software) of ATP6V1A and indicated, previously reported STAT3 binding partners in anti-STAT3 and mIgG2a control IPs analyzed by nanoLC-MS/MS. Error bars, SD of three independent experiments.
c Representative immunoblots verifying the STAT3 depletion in H6C7 STAT3-KO pool and HMF3-STAT3-KO clones. HMF3-STAT3-KO-1 clone was used in Figure 2g and 4b. N = 3.
