# Supplementary material for: STAT3 associates with vacuolar H+-ATPase and regulates cytosolic and lysosomal pH
Source: Cell Res. 2018 Aug 20;28(10):996–1012. doi: 10.1038/s41422-018-0080-0 (PMC6170402; doi:10.1038/s41422-018-0080-0)
Supplement: Supplementary file 3 — Supplementary information, Figure S3 [file 41422_2018_80_MOESM3_ESM.pdf]

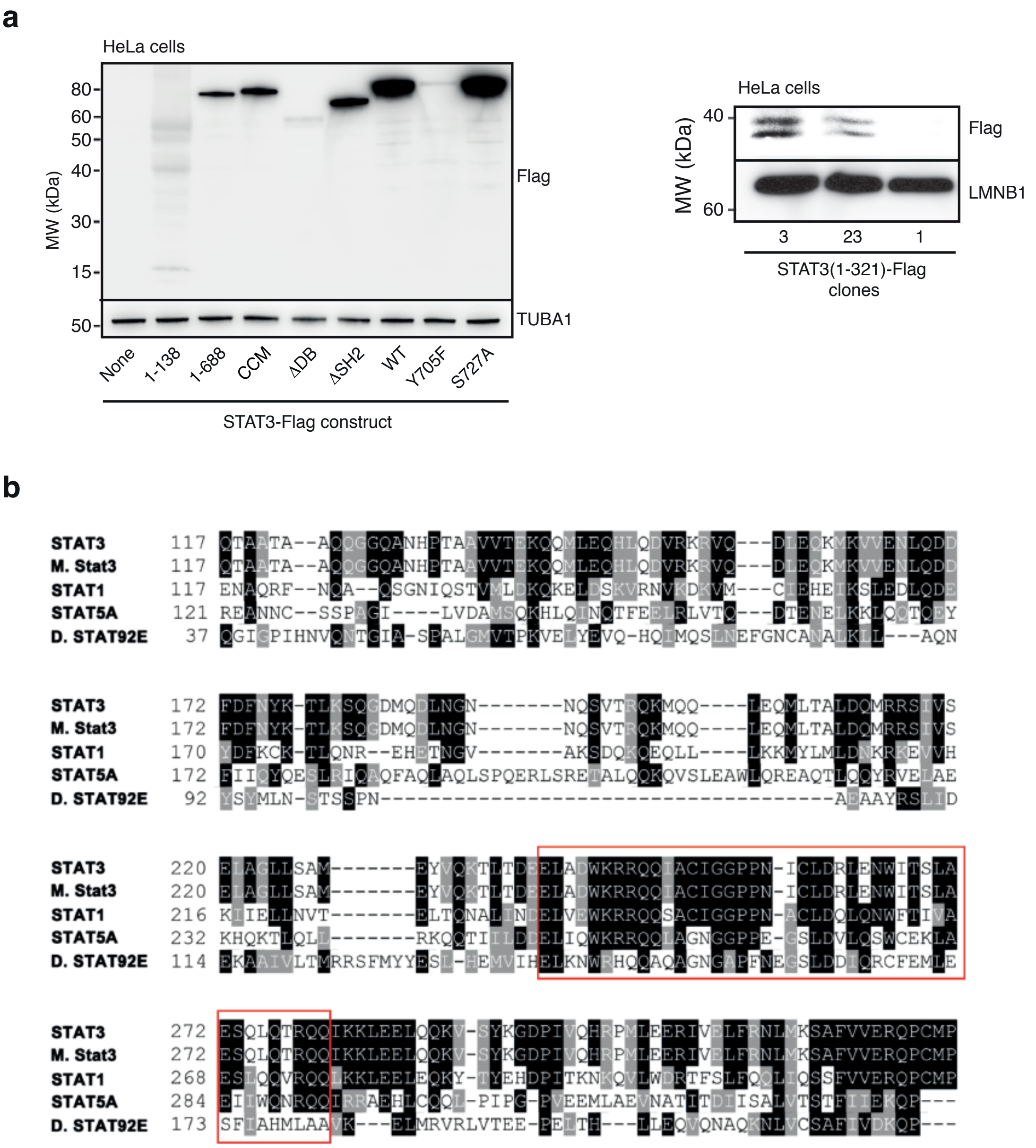

Figure S3. STAT3 interacts with V-ATPase via its coiled coil domain
a Alignment of coiled coil domain sequences of human STAT3, murine STAT3, human STAT1, human STAT5A and drosophila STAT92E. The red box indicates the sequence deleted in STAT3-CMM used in Figures 3b and Supplementary Figure S4b.
b Representative immunoblots of indicated STAT3-Flag constructs in total cell lysates of HeLa single cell clones. The blot on the right was exposed for longer time in order to detect the low expression of STAT3(1-321)-Flag protein. Clone 23 was used in the experiments presented in Figure 3b.
