# Supplementary material for: STAT3 associates with vacuolar H+-ATPase and regulates cytosolic and lysosomal pH
Source: Cell Res. 2018 Aug 20;28(10):996–1012. doi: 10.1038/s41422-018-0080-0 (PMC6170402; doi:10.1038/s41422-018-0080-0)
Supplement: Supplementary file 4 — Supplementary information, Figure S4 [file 41422_2018_80_MOESM4_ESM.pdf]

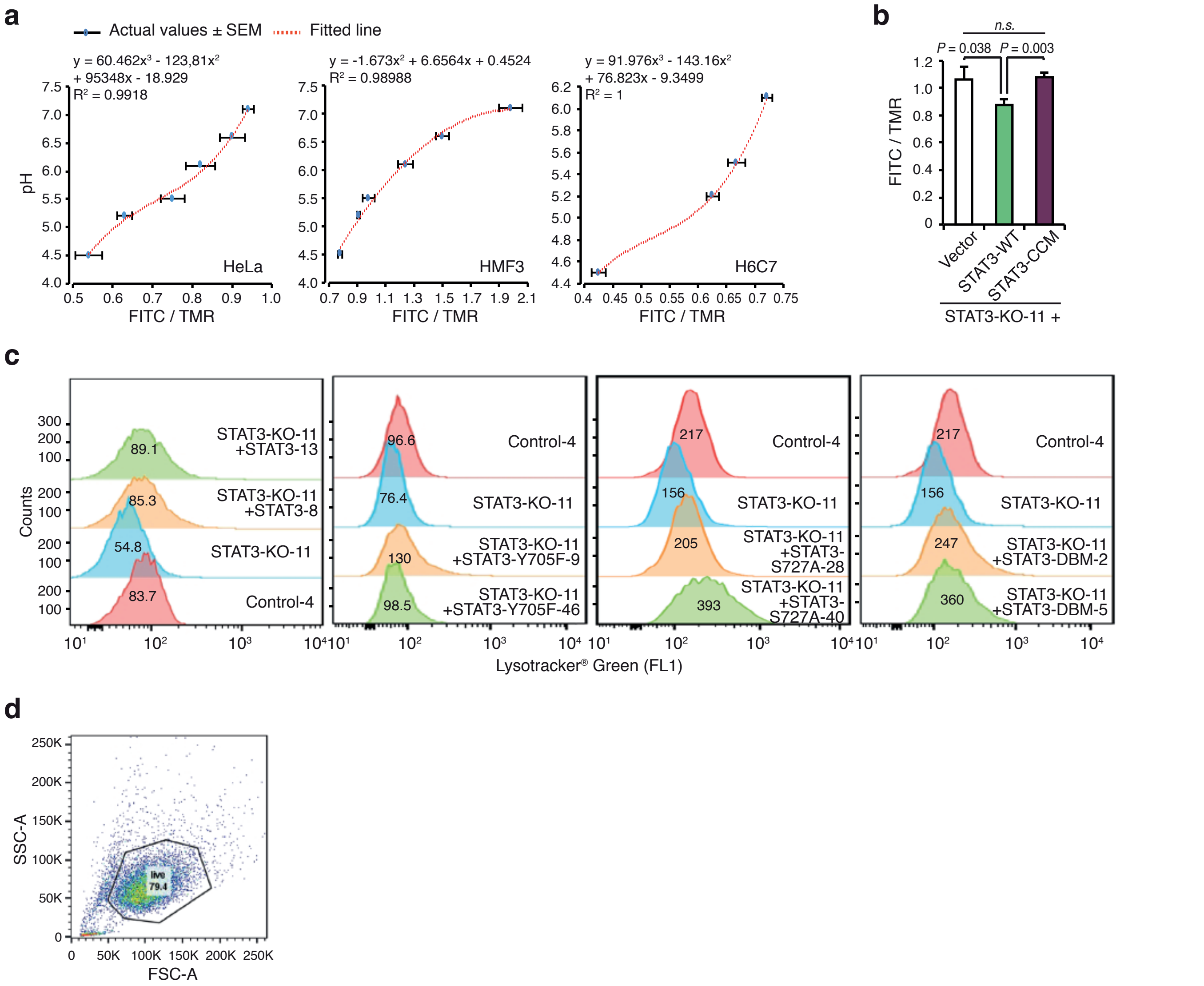

Figure S4. STAT3 regulates lysosomal pH

a Standard curves for lysosomal pH measurements in HeLa, HMF3 and H6C7 cells presented in Figures 4a and b. Error bars, SEM of three measurements.

b FITC/TMR ratio that reflects the lysosomal pH in HeLa cells transiently transfected with indicated STAT3 constructs. Error bars, SD of three independent experiments with ≥10 cells / sample were analyzed. P values were calculated by 2-tailed, homoscedastic student's t-test.

c Representative flow cytometer profiles showing volumes of acidic compartment (VAC) in a HeLa CRISPR control cell clone (C-4), STAT3-KO clones (KO-1 and -11), and KO-11 clone resubstituted with wild type (WT) or mutated (Y705F, DBM, S727A) STAT3 constructs. Cells were analyzed by flow cytometer after 5 min staining with 75 nM Lysotracker® Green followed by a wash in PBS. Quantification of 3 independent experiments is shown in Figure 4c.

d Gating of the cells in flowcytometry analysis. SSC-A, side-scattered light; FSC-A, forward-scattered light.
