# Supplementary material for: STAT3 associates with vacuolar H+-ATPase and regulates cytosolic and lysosomal pH
Source: Cell Res. 2018 Aug 20;28(10):996–1012. doi: 10.1038/s41422-018-0080-0 (PMC6170402; doi:10.1038/s41422-018-0080-0)
Supplement: Supplementary file 5 — Supplementary information, Figure S5 [file 41422_2018_80_MOESM5_ESM.pdf]

a

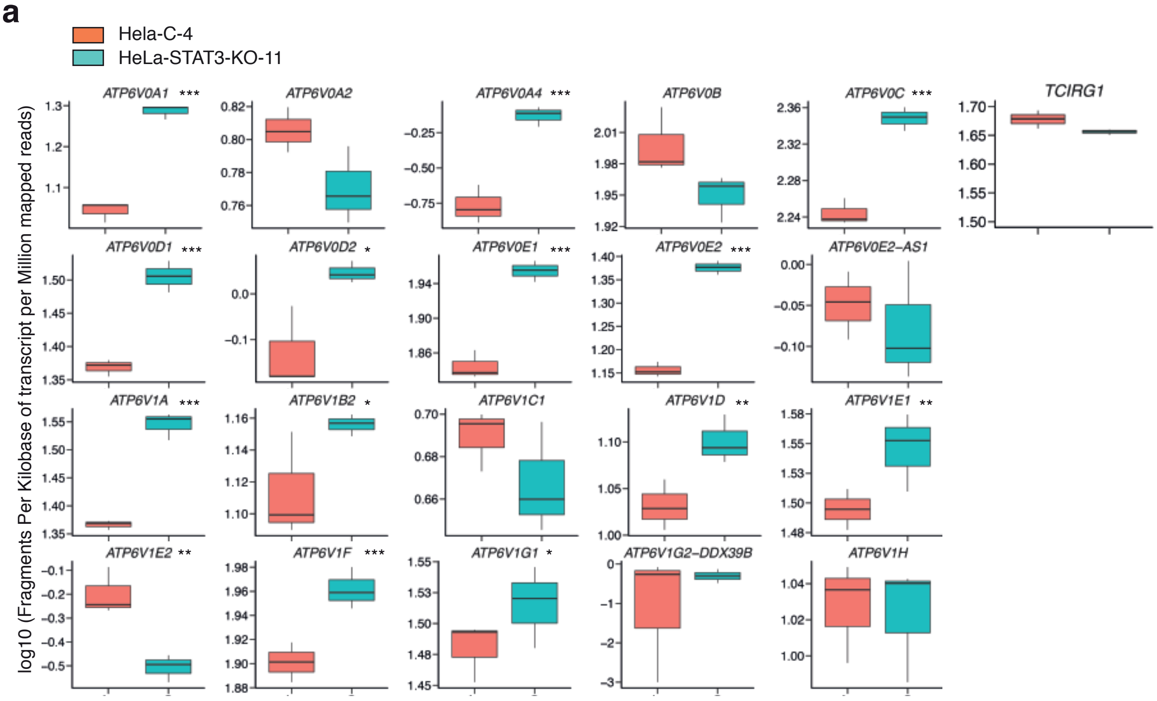

b

	Gene	Normalized counts WT	Normalized counts STAT3-KO	log2 fold change STAT3-KO / WT	*P* value
Down in STAT3-KO	*ABCA2*	14232	8389	-0.76	1.10E-78
	CTSH	91	3842	-1.25	6.57E-07
	GAA	1536	751	-1.03	4.30E-48
	MANBA	1156	734	-0.66	4.48E-19
	GALC	109	48	-1.19	1.84E-07
	CTSZ	7103	3787	-0.91	5.01E-67
	CTSC	10689	6389	-0.74	2.82E-64
	CTSF	1740	824	-1.08	1.11E-56
	HYAL1	1559	457	-1.77	6.83E-116
	IDUA	349	200	-0.80	1.399-07
	HEXB	5762	2909	-0.99	2.96E-95
	HLA-DRA	28	12	-1.18	0.0061
	RAMP2	15	6	-1.29	0.0141
Up in STAT3-KO	*ABCB9*	204	453	1.15	3.82E-22
	ADRB2	217	329	0.60	2.55E-06
	AP1M2	171	441	1.36	2.06E-22
	ATP6V0A1	1406	2512	0.84	3.92E-41
	ATP6V0A4	17	70	2.03	3.53E-10
	ATP6V0D2	54	81	0.59	0.016882
	CLTC	11840	18044	0.61	1.19E-53
	NEU1	588	898	0.61	8.21E-14
	SORT1	451	1150	1.35	1.63E-63
	TOM1	481	782	0.70	2.00E-15

c

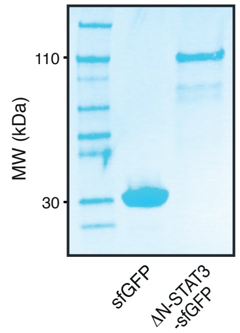

Figure S5. STAT3 does not regulate the expression of V-ATPse subunits.
a Box plot of expression (Fragments Per Kilobase of transcript per Million mapped reads) of indicated mRNAs encoding all known V-ATPase subunits and related non-coding RNAs in HeLa CRISPR control (C-4) and STAT3-KO (KO-11) cells defined by RNASeq of three independent samples for each cell line. from 25% quantile to 75% quantile and median in the middle). Horizontal line, median; lower hinge, 25% quantile; upper hinge, 75% quantile; lower whisker, observation greater than or equal to lower hinge - 1.5 * IQR (interquartile range); upper whisker, largest observation less than or equal to upper hinge + 1.5 * IQR.
b Expression of genes whose protein products according to either Gene Ontology (http://www.geneontology.org/page/go-enrichment-analysis) or Kyoto Encyclopedia of Genes and Genomes (www.genome.jp/kegg/pathway.html) databases localize to lysosomes, and whose expression was over ≥ 1.5-fold (P ≤ 0.05) altered in HeLa-STAT3-KO cells as compared to CRISPR control cells. Expression levels were defined by RNASeq. P values were defined by DEseq2.
c Coomassie-stained protein gel visualizing indicated recombinant proteins produced in E.coli and used in Figure 5f.
