# Supplementary material for: STAT3 associates with vacuolar H+-ATPase and regulates cytosolic and lysosomal pH
Source: Cell Res. 2018 Aug 20;28(10):996–1012. doi: 10.1038/s41422-018-0080-0 (PMC6170402; doi:10.1038/s41422-018-0080-0)
Supplement: Supplementary file 6 — Supplementary information, Figure S6 [file 41422_2018_80_MOESM6_ESM.pdf]

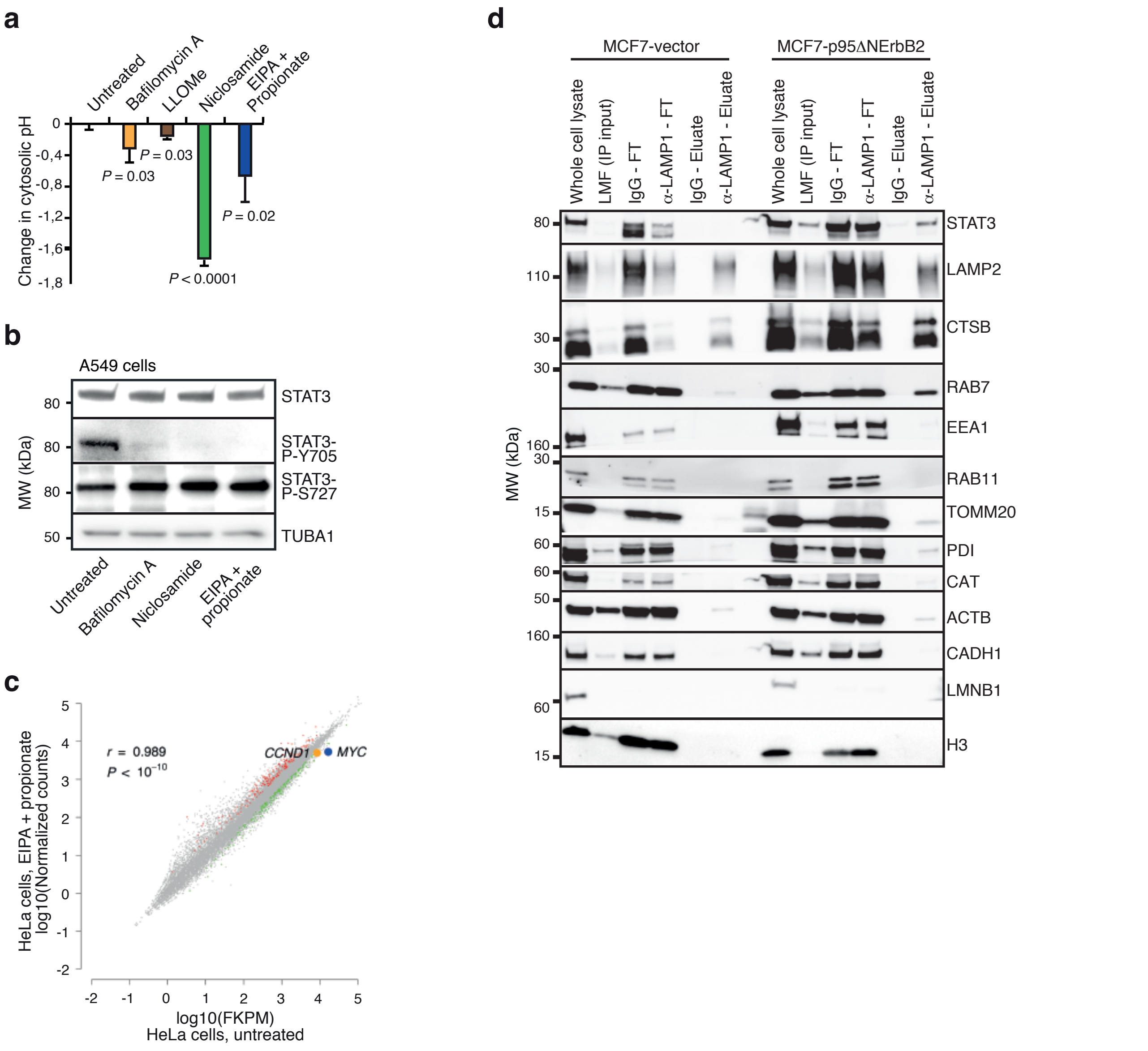

Figure S6. Regulation of STAT3 by cytosolic acidification

a Cytosolic pH of HeLa cells left untreated or treated with 0.1 μM bafilomycin A1, 1 mM LLOMe or 10 μM niclosamide for 1 h or 25 μM EIPA + 50 mM propionate for 0.5 h was determined by confocal image analyses of cells loaded with pHrodo™ Green AM. Values are expressed as change in pH compared to untreated cells. Error bars, - SD of three independent experiments. P values were calculated by 2-tailed, homoscedastic student's t-test.

b Representative immunoblots of indicated proteins in lysates of A549 cells treated with 0.1 μM bafilomycin A1 or 10 μM niclosamide for 1h or 25 μM EIPA + 50 mM propionate for 30 min.

c Three independent RNA samples from HeLa cells left untreated or treated with 25 μM EIPA + 50 mM propionate for 4 h were analyzed by RNA Seq. Out of the 2001 putative direct STAT3 target genes identified previously by Chip sequencing (see materials and methods for details), 1630 were detected and their expression levels are indicated in the graph. Genes significantly downregulated or upregulated are marked green and red, respectively. Moreover, mRNAs for two well-characterized STAT3 target genes that were significantly downregulated by the treatment, CCND1 and MYC, are marked orange and blue, respectively.

d Representative immunoblots of STAT3 and indicated organelle markers in total cell lysates, light membrane fractions (LMF) or indicated flow throughs (FT) and immunoprecipitates (eluate) from MCF7-vector and MCF7-p95DNErbB2. N = 4.
