# Supplementary material for: STAT3 associates with vacuolar H+-ATPase and regulates cytosolic and lysosomal pH
Source: Cell Res. 2018 Aug 20;28(10):996–1012. doi: 10.1038/s41422-018-0080-0 (PMC6170402; doi:10.1038/s41422-018-0080-0)
Supplement: Supplementary file 7 — Supplementary information, Figure S7 [file 41422_2018_80_MOESM7_ESM.pdf]

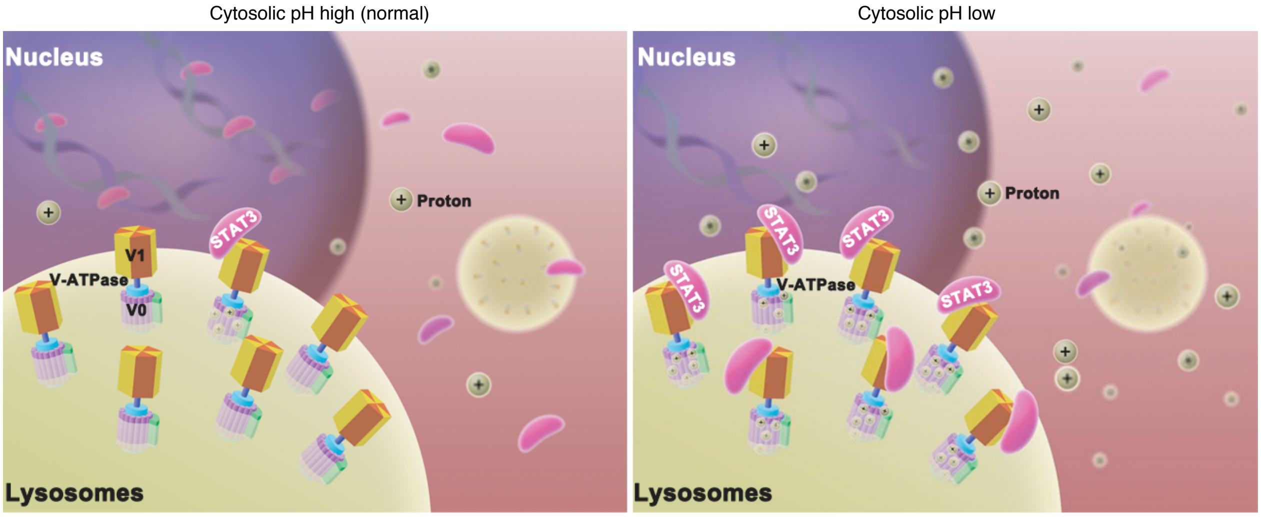

Figure S7. Lysosomal STAT3 regulates intracellular pH, which in turn regulates STAT3 localization and activity
Schematic presentation of the regulation of the lysosomal V-ATPase by STAT3. When cytosolic pH is high in normal growth conditions, a small proportion of cellular STAT3 is associated with the V-ATPase ensuring the maintenance of alkaline cytosolic pH. Upon acidification of the cytosol, *e.g.* due to increased glycolysis, more STAT3 is recruited to the lysosomal membrane to enhance the V-ATPase activity.
