# Supplementary material for: STAT3 associates with vacuolar H+-ATPase and regulates cytosolic and lysosomal pH
Source: Cell Res. 2018 Aug 20;28(10):996–1012. doi: 10.1038/s41422-018-0080-0 (PMC6170402; doi:10.1038/s41422-018-0080-0)
Supplement: Supplementary file 8 — Supplementary information, Table S1 [file 41422_2018_80_MOESM8_ESM.pdf]

Table S1. Reagents and resources

REAGENT	SOURCE	IDENTIFIER
**Antibodies**		
ACTB, mouse, HRP-coupled	Abcam	Cat# ab20272
ATP6V0A4, rabbit	Abcam	Cat# ab97440
ATP6V0D1, mouse	Abcam	Cat# ab56441
ATP6V0D1, rabbit	Abcam	Cat# ab202899
ATP6V1A, mouse	Sigma-Aldrich	Cat# SAB1402125
ATP6V1A, rabbit	Abcam	Cat# ab199326
ATP6V1A, rabbit	Abcam	Cat# ab137574
ATP6V1B2, rabbit	Abcam	Cat# ab73404
CAT, mouse	Abcam	Cat# ab16771
CD63, mouse	Devel. Studies Hybridoma Bank	Clone H5C6
CTSB, mouse	Provided by Ekkehard Weber	Ref. # [1]
CTSD, mouse	BD Transduction Laboratories™	Cat# 610800
Cyclin D, rabbit	Millipore	Cat# 06-137
EEA1, rabbit	Abcam	Cat# ab2900
FLAG®, mouse	Sigma-Aldrich	Cat# F1804
FLAG®, mouse, HRP-coupled	Sigma-Aldrich	Cat# A8592
FLAG®, mouse, magnetic bead	Sigma-Aldrich	Cat# M8823
GRP75, mouse	Enzo Life Sciences	Cat# ADI-SPS-825-D
Guinea pig IgG, peroxidase-conjugated	DAKO	Cat# P0141
Hemagglutinin, mouse	Sigma-Aldrich	Cat# H3663
Hemagglutinin, rabbit, magnetic bead	Cell Signaling	Cat# 11846
Histone H3, rabbit	Abcam	Cat# ab18521
IgG, mouse	Cell Signaling	Cat# 5873S
IgG, mouse, peroxidase-conjugated	DAKO	Cat# P0260
IgG, rabbit	Cell Signaling	Cat# 8726S
LAMP1, mouse	Santa Cruz Biotechnology	Cat# sc-20011
LAMP1, rabbit	Abcam	Cat# ab24170
LMNB1, rabbit, horseradish peroxidase (HRP)-coupled	Abcam	Cat# ab194109
Mouse IgG, donkey, Alexa Fluor® 488-conjugated	Thermo Fisher Scientific	Cat# A-21202
PDI, rabbit	Abcam	Cat# ab 3672
RAB5A, rabbit	Santa Cruz Biotechnology	Cat# sc-309
RAB7, goat	Santa Cruz Biotechnology	Cat# sc-6563
RAB11, rabbit	Abcam	Cat# ab3612
Rabbit IgG, donkey, Alexa Fluor® 568-conjugated	Thermo Fisher Scientific	Cat# A-10042
Rabbit IgG, goat, micro bead-conjugated	Miltenyl Biotec	Cat# 130-048-602
Rabbit IgG, peroxidase-conjugated	Vector Laboratories	Cat# PI-1000
RTN3, mouse	Santa Cruz Biotechnology	Cat# sc-374599
STAT3, mouse	Cell Signaling	Cat# 9139

STAT3, mouse	BD Transduction Laboratories™	Cat# 610189
STAT3, rabbit	Cell Signaling	Cat# 4904
STAT3, rabbit	Cell Signaling	Cat# 12640
STAT3 P-S727-, rabbit	Abcam	Cat# ab86430
STAT3 P-Y705, rabbit	Cell Signaling	Cat# 9145
STAT3 with non-phosphorylated Ser-727, rabbit	Abcam	Cat# ab32500 P-
Survivin, mouse	Santa Cruz Biotechnology	Cat# sc-17779
TOMM20, mouse	Santa Cruz Biotechnology	Cat# sc-11415
TUBA1A, mouse, HRP-coupled	Abcam	Cat# ab40742
VDAC, rabbit	Cell Signaling	Cat# 12454s
**Bacterial Strains**		
DH5α *Escherichia coli*	Thermo Fisher Scientific	Cat# 11319-019
BL21 Star™(DE3) *E.coli*	Thermo Fisher Scientific	Cat# C6010-03
**Chemicals, Peptides, and Recombinant Proteins**		
5-(N-Ethyl-N-isopropyl) amiloride (EIPA)	Sigma-Aldrich	Cat# A3085
Benzonase® Nuclease	Sigma-Aldrich	Cat# E1014-5KU
Bromophenol blue	Sigma-Aldrich	Cat# B0126
Digitonin	Sigma-Aldrich	Cat# D141
Dithiothreitol	Sigma-Aldrich	Cat# D9779
Hoechst 33342	Sigma-Aldrich	Cat# B2261
Glutamine	Sigma-Aldrich	Cat# G7513
Niclosamide	Sigma-Aldrich	Cat# N3510
Nigericin sodium salt	Sigma-Aldrich	Cat# N7143
Oligomycin A	Sigma-Aldrich	Cat# 75351
Propionic acid	Sigma-Aldrich	Cat# 402907
Puromycin	Sigma-Aldrich	Cat# P7255
Protamine sulfate salt	Sigma-Aldrich	Cat# P4020
Saponin	Sigma-Aldrich	Cat# S4521
Trypan blue	Sigma-Aldrich	Cat# T8154
CHAPS	Sigma-Aldrich	Cat# C5070
Valinomycin	Sigma-Aldrich	Cat# V0627
Triton-X-100	Sigma-Aldrich	Cat# T9284
Tween-20	Sigma-Aldrich	Cat# 274348
XtremeGENE™ 9 DNA transfection reagent	Sigma-Aldrich	Cat# XTG9-RO
10 kDa dextran coupled to cascade blue	Thermo Fisher Scientific	Cat# D1976
70 kDa dextran coupled to fluorescein/tetramethylrodamine	Thermo Fisher Scientific	Cat# D1951
Alexa Fluor® 488	Thermo Fisher Scientific	Cat# D22910
AlexaFluor® 594	Thermo Fisher Scientific	Cat# D22913
Dithiobis(succinimidyl propionate) (DSP)	Thermo Fisher Scientific	Cat# 22585
DMEM	Thermo Fisher Scientific	Cat# 31966-021
Dulbecco's phosphate-buffered saline (DPBS)	Thermo Fisher Scientific	Cat# 14190-094
Keratinocyte-serum free media with supplements (SFM)	Thermo Fisher Scientific	Cat# 17005042
Lipofectamine® 3000 Transfection Reagent	Thermo Fisher Scientific	Cat# L3000008

Lipofectamine® RNAmax	Thermo Fisher Scientific	Cat# 13778075
Live Cell Imaging Solution	Thermo Fisher Scientific	Cat# A14291DJ
Lysotracker® Green DND-26	Thermo Fisher Scientific	Cat# L7526
Octyl-ß-glucoside	Thermo Fisher Scientific	Cat# 28310
PepClean C-18 Spin columns	Thermo Fisher Scientific	Cat# 89870
PepMap EASY-spray	Thermo Fisher Scientific	Cat# ES802
pHrodo™ Green AM Intracellular pH Indicator	Thermo Fisher Scientific	Cat# P35373
PowerLoad™ concentrate	Thermo Fisher Scientific	Cat# P10020
Fetal calf serum	Life Technologies	Cat# 10270-106
G418	Life Technologies	Cat# 11811-031
Penicillin/streptomycin	Life Technologies	Cat# 15140-122
Prolong Gold Antifade mounting medium with DAPI	Life Technologies	Cat# P36935
Bafilomycin A1	Invivogen	Cat# tlrl-baf1
Normocin™	Invivogen	Cat# ant-nr-1
Protease inhibitor cocktail	Roche	Cat# 11697498001
Protease inhibitor cocktail	Roche	Cat# 04693159001
Phosphatase inhibitor cocktail	Roche	Cat# 04906837001
BIOMOL® Green Reagent	Enzo Life Sciences	Cat# BML-AK111
Hygromycin B	Calbiochem	Cat# 400051
Clarity Western ECL Substrate	Bio-Rad	Cat# 170-5061
Paraformaldehyde (PFA)	Ampliqon	Cat# 43226.1000
Methanol	Fluka	Cat# 34966)
Bovine serum albumin (BSA)	Amresco	Cat# E531
Goat serum	DAKO	Cat# X0907
TurboFectin 8.0 transfection agent	Origene	Cat# TF81005
PEG-it™ Virus Precipitation Solution	System Biosciences, Inc.	Cat# LV810A-1
BugBuster® 10X Protein Extraction Reagent	Merck Millipore	Cat# 70921
KOD Xtreme™ Hot Start DNA Polymerase	Merck Millipore	Cat# 71975
Ni-NTA affinity resins	Qiagen	Cat# 30210
**Commercial Assays**		
Duolink® In Situ Red Starter Kit	Sigma-Aldrich	Cat# DUO92101
Intracellular pH Calibration Buffer Kit	Thermo Fisher Scientific	Cat# P35379
SuperScript™ III Platinum™ SYBR™ Green One-Step qPCR Kit w/ROX	Thermo Fisher Scientific	Cat# 11746100
Venor®GeM Classic PCR kit	Minerva Biolabs	Cat# 11-1100
**Cell Lines**		
Cell Line: A549	ATCC	CRM-CCL-185™
Cell Line: HeLa	ATCC	CRM-CCL-2™
Cell Line: SKOV3	ATCC	HTB-77™
Cell Line: H6C7	Kerafast	Cat# ECA001
	Cell Line: HMF3	
	Michael J. O'Hare, Ludwig	Ref. # [2]

	Institute, London, UK)	
Cell Line: Lenti-X™ 293T	Clontech	632180
	Cell Line: A549-triple	
	Dmitry Malkov, Sigma-Aldrich	Ref. # [3]

Recombinant DNA

pAAV-MSC	Agilent Technologies	Cat# 240071
pBCMV-MCS-puro	System Biosciences	Cat# PB510B-1
pEGFPN1	Clontech	Cat# 632469
pPACKH1 HIV Lentivector Packaging Kit	System Biosciences	Cat# LV500A-1
pCDH-CMV-MCS-EF1-Hygro	System Biosciences	Cat# CD515B-1
pCDNA3.1-DYK	Genscript	Cat# 644249_1
pCDNA3.1-DYK-STAT3	Genscript	Custom made
pCDNA3.1-HA-ATP6V1A	Genscript	Custom made
Super transposase expression vector	System Biosciences	Cat# PB210PA-1
ptfLC3	Dr. T. Yoshimori Ref. # [4]	Addgene ID:21074
EF.STAT3-Y705F.Ubc.GFP	Dr. Linzhao Cheng Ref. # [5]	Addgene ID: 24983
mTagBFP2-MannII-N-10	Dr. Michael Davidson Ref. # [6]	Addgene ID: 55309
mTagBFP2-Rab5a-7	Dr. Michael Davidson Ref. # [6]	Addgene ID: 55322
mito-BFP	Dr. Gia Voeltz Ref. # [7]	Addgene ID: 49151
BFP-KDEL	Dr. Gia Voeltz Ref. # [7]	Addgene ID: 49150
pMA-SpCas9-g1	Dr. Yonglun Luo Ref. # [8]	Addgene ID: 80784
pMA-SpCas9-g2	Dr. Yonglun Luo Ref. # [8]	Addgene ID: 80785
pxpr001	Dr. Feng Zhang Ref. # [9]	N/A
pSpCas9(BB)-2A-Puro	Dr. Feng Zhang Ref. # [10]	N/A
pNeDaKo-Neo	Dr. Bert Vogelstein	N/A
pETM11SUMO3sfGFP	Dr. Anne-Claude Gavin	N/A
pETM11SUMO3ΔNSTAT3sfGFP	This paper	N/A
pBCMV-MCS-puro-STAT3-flag	This paper	N/A
pCDH-hygroF	This paper	N/A
pCDH-hygroF-STAT3	This paper	N/A
pCDH-hygroF-STAT3-Y705F	This paper	N/A
pCDH-hygroF-STAT3-DBM	This paper	N/A
pCDH-hygroF-STAT3-S727A	This paper	N/A

pCDH-hygroF-STAT3(1-138)-flag	This paper	N/A
pCDH-hygroF-STAT3(1-321)-flag	This paper	N/A
pCDH-hygroF-STAT3(1-496)-flag	This paper	N/A
pCDH-hygroF-STAT3(1-688)-flag	This paper	N/A
pCDH-hygroFSTAT3-S727A-flag	This paper	N/A
pBFPN1	This paper	N/A
pLAMP1-BFP	This paper	N/A
pLAMP2-BFP	This paper	N/A
pAAV-EGFP-MMEJ	This paper	N/A
pMA-STAT3-gRNA	This paper	N/A
pMA-MMEJ-gRNA	This paper	N/A
pCDNA3.1DYK-STAT3delta239-280	This paper	N/A
pCDNA3.1DYK-STAT3deltaCC	This paper	N/A
pCDNA3.1DYK-STAT3deltaSH2	This paper	N/A
pCDNA3.1DYK-STAT3deltaDB	This paper	N/A
**Sequence-based reagents**		
All Star non-targeting control siRNA	Qiagen	Cat# SI03650318
ATP6V1A siRNA: 5'-ACAGAGGAACUGUAACUUA	Eurofins Genomics	Custom made
ATP6V0D1 siRNA: 5'-CUGGCUCGGGCUGACGACUAU	Eurofins Genomics	Custom made
STAT3 sgRNA primers:<br>5'-CACCGGCAGCTTGACACACGGTACC<br>5'-AAACGGTACCGTGTGTCAAGCTGC	Eurofins Genomics	Custom made
STAT3 quide sequences:<br>Cr-Uni: 5'- GGCCATCCTGCTAAAATCAG<br>Cr-STAT3: 5'- TCCATCACTAGGGGTTCCTG	Eurofins Genomics	Custom made
qPCR primers for *CCND1*:<br>Primer 1: 5'-GAAGATCGTCGCCACCTG<br>Primer 2: 5'-GACCTCCTCCTCGCACTTCT	Eurofins Genomics	Custom made
qPCR primers for *ACTB*:<br>Primer 1: 5'-TGACTTAGTTGCGTTACACCCTT<br>Primer 2: 5'-CACCTTCACCGTTCCAGTTTT	Eurofins Genomics	Custom made
Primers for site-specific mutagenesis	See Table S1	N/A
Primers for subcloning	See Table S2	N/A
**Softwares and Algorithms**		
Flow Jo	FLOWJO LLC	www.flowjo.com/solutions/flowjo/downloads
Image J (Fiji)	IMAGEJ	imagej.net/Fiji
Image Studio Lite software	LI-COR Biosciences	www.licor.com/bio/products/software/image_studio_lite/
MaxQuant software version 1.5.2.8	Ref. # [11] N/A	
Prism7	GraphPad	www.graphpad.com/scientific-software/prism/
ZEN 2010 software	Carl Zeiss	N/A

ZEN software black edition 2012 (Super resolution)	Carl Zeiss	N/A
**Others**		
Agilent 2100 BioAnalyzer	Agilent Technologies	N/A
BD FACSVerse™	BD Biosciences	N/A
Bio-Rad Trans-Blot Turbo system	BioRad	Cat# 1704156<br>Cat# 1704157
Celigo® Cell Imaging Cytometer	Nexcelom Bioscience	N/A
EASY nanoLC coupled to a Q Exactive Plus Hybrid Quadrupole-Orbitrap Mass Spectrometer	Thermo Fisher Scientific	N/A
Luminescent Image Reader	Fujifilm	LAS-4000
LS MACS™ separation columns	Miltenyl Biotec	Cat# 130-042-401
OctoMACS™ Dissociator	Miltenyl Biotec	Cat# 130-095-937
OctoMACS™ Separator	Miltenyl Biotec	Cat# 130-042-109
Varioskan® Flash Multimode Reader	Thermo Fisher Scientific	N/A
Elyra PS.1 microscope	Carl Zeiss	N/A
sCMOS PCO.edge camera	PCO	N/A
Plan-Apochromat 63x/1.40 Oil DIC M27 objective	Carl Zeiss	N/A
Zeiss LSM700 microscope	Carl Zeiss	N/A
63x, 1.4NA, oil immersion objective lens	Carl Zeiss	N/A

SUPPLEMENTARY REFERENCES

1 Weber, E. *et al.* Cathepsin B-deficient mice as source of monoclonal anti-cathepsin B antibodies. *Biol Chem* **396**, 277-281, doi:10.1515/hsz-2014-0191 (2015).
2 O'Hare, M. J. *et al.* Conditional immortalization of freshly isolated human mammary fibroblasts and endothelial cells. *Proc Natl Acad Sci U S A* **98**, 646-651, doi:10.1073/pnas.98.2.646 (2001).
3 Samsonov, A. *et al.* Tagging of genomic STAT3 and STAT1 with fluorescent proteins and insertion of a luciferase reporter in the cyclin D1 gene provides a modified A549 cell line to screen for selective STAT3 inhibitors. *PLoS One* **8**, e68391, doi:10.1371/journal.pone.0068391 (2013).
4 Kimura, S., Noda, T. & Yoshimori, T. Dissection of the autophagosome maturation process by a novel reporter protein, tandem fluorescent-tagged LC3. *Autophagy* **3**, 452-460 (2007).
5 Hillion, J. *et al.* The high-mobility group A1a/signal transducer and activator of transcription-3 axis: an achilles heel for hematopoietic malignancies? *Cancer Res* **68**, 10121-10127, doi:10.1158/0008-5472.CAN-08-2121 (2008).
6 Subach, O. M., Cranfill, P. J., Davidson, M. W. & Verkhusha, V. V. An enhanced monomeric blue fluorescent protein with the high chemical stability of the chromophore. *PLoS One* **6**, e28674, doi:10.1371/journal.pone.0028674 (2011).
7 Friedman, J. R. *et al.* ER tubules mark sites of mitochondrial division. *Science* **334**, 358-362, doi:10.1126/science.1207385 (2011).
8 Vad-Nielsen, J., Lin, L., Bolund, L., Nielsen, A. L. & Luo, Y. Golden Gate Assembly of CRISPR gRNA expression array for simultaneously targeting multiple genes. *Cell Mol Life Sci* **73**, 4315-4325, doi:10.1007/s00018-016-2271-5 (2016).
9 Shalem, O. *et al.* Genome-scale CRISPR-Cas9 knockout screening in human cells. *Science* **343**, 84-87, doi:10.1126/science.1247005 (2014).
10 Ran, F. A. *et al.* Double nicking by RNA-guided CRISPR Cas9 for enhanced genome editing specificity. *Cell* **154**, 1380-1389, doi:10.1016/j.cell.2013.08.021 (2013).
11 Cox, J. & Mann, M. MaxQuant enables high peptide identification rates, individualized p.p.b.-range mass accuracies and proteome-wide protein quantification. *Nat Biotechnol* **26**, 1367-1372, doi:10.1038/nbt.1511 (2008).
