# Supplementary material for: STAT3 associates with vacuolar H+-ATPase and regulates cytosolic and lysosomal pH
Source: Cell Res. 2018 Aug 20;28(10):996–1012. doi: 10.1038/s41422-018-0080-0 (PMC6170402; doi:10.1038/s41422-018-0080-0)
Supplement: Supplementary file 9 — Supplementary information, Table S2 [file 41422_2018_80_MOESM9_ESM.pdf]

Table S2. Primers used for site-directed mutagenesis of *STAT3.*

Mutations	Primer 1	Primer 2
S727A	CCATT GACCT GCCGA TGGCC CCCCG CACTT TAGAT TC	GAATC TAAAG TGCGG GGGGC CATCG GCAGG TCAAT GG
DBM V461-463A	CCTAG AGACC CACTC CTTGC CAGCT GCGGC GATCT CCAAC ATCTG TCAGA TG	CATCT GACAG ATGTT GGAGA TCGCC GCAGC TGGCA AGGAG TGGGT CTCTA GG
DBM E434-435A	GATGC TTCCC TGATT GTGAC TGCGG CGCTG CACCT GATCA CCTTT GAG	CTCAA AGGTG ATCAG GTGCA GCGCC GCAGT CACAA TCAGG GAAGC ATC
ΔDB	GCCTTT CCAATTG*G*A*A*CCTGGGATCAAG	AATTGG AAAGGC A*C*T*T*TTCATTAAGTTTC
Δ239-280	CGGACGAGGAG ATTAAGAAAC TGGAGGAGTTGCAGCAAAAAGTTTC	GTTTCTTAATCTCCTCGTCCG TGAGAGTTTTCTGCACGTACTCCAT
ΔSH2	TACATC CCAGAGA*G*C*C*AGGAGCATCCTG	CTCTGG GATGTAC*C*C*T*TCGTTCCAAAG
