# Supplementary material for: STAT3 associates with vacuolar H+-ATPase and regulates cytosolic and lysosomal pH
Source: Cell Res. 2018 Aug 20;28(10):996–1012. doi: 10.1038/s41422-018-0080-0 (PMC6170402; doi:10.1038/s41422-018-0080-0)
Supplement: Supplementary file 10 — Supplementary information, Table S3 [file 41422_2018_80_MOESM10_ESM.pdf]

Table S3. Construction of expression vectors.

Construct	Vector	Primer 1	Primer 2	Cloning sites	Template
pBCMV-MCS-puro-STAT3-flag, STAT3-Y705F-flag	pBCMV-MCS-puro	gc GCTAGC cacc ATGGCCCAATGG AATCAGCTAC	gc TTCGAA tca cttatcgtcgtcatccttgta atc CATGGGGGAGGT AGCGCACTCCGA G	NheI, BstBI	pCDNA3.1-DYK-STAT3, EF.STAT3-Y705F.Ubc.GFP
pCDH-hygroFinal (pCDH-hygroF)	pCDH-CMVhygro	CTAGC gatatcctcgagcccggg atgcataccggtg	GATCC accggtatgcatcccggg ctcgaggatatcg	NheI, BamHI	N/A
pCDH-hygroF-STAT3, STAT3-Y705F, STAT3-S727A	pCDH-hygroF	gc GCTAGC cacc ATGGCCCAATGG AATCAGCTAC	gc ACCGGTTCACAT GGGGGAGG TAGCGCACTC	NheI, AgeI	Ouick change vectors got from table S1
pCDH-hygroF-STAT3(1-138)-flag	pCDH-hygroF	gc GCTAGC cacc ATGGCCCAATGG AATCAGCTAC	GC ACCGGT tca cttatcgtcgtcatccttgta atcCGTCACCACG GCTGCTGTGGG	NheI, AgeI	pCDNA3.1-DYK-STAT3
pCDH-hygroF-STAT3(1-321)-flag	pCDH-hygroF	gc GCTAGC cacc ATGGCCCAATGG AATCAGCTAC	GC ACCGGT tca cttatcgtcgtcatccttgta atc AAAGGCACTTTTC ATTAAGTT	NheI, AgeI	pCDNA3.1-DYK-STAT3
pCDH-hygroF-STAT3(1-496)-flag	pCDH-hygroF	gc GCTAGC cacc ATGGCCCAATGG AATCAGCTAC	GC ACCGGT tca cttatcgtcgtcatccttgta atc GGGCTTGGTAAA AAAGTTTAC	NheI, AgeI	pCDNA3.1-DYK-STAT3
pCDH-hygroF-STAT3(1-688)-flag	pCDH-hygroF	gc GCTAGC cacc ATGGCCCAATGG AATCAGCTAC	GC ACCGGT tca cttatcgtcgtcatccttgta atc CCGACAATACTTT CCGAATGC	NheI, AgeI	pCDNA3.1-DYK-STAT3
pCDH-hygroF-STAT3-Y705F, STAT3-S727A,	pCDH-hygroF	gc GCTAGC cacc ATGGCCCAATGG AATCAGCTAC	GC ACCGGT tca cttatcgtcgtcatccttgta atc CATGGGGGAGGT AGCGCACTC	NheI, AgeI	Ouick change vectors got from table S1
pBFPN1	pEGFPN1	gc GGATCC ATGAGCGAGCTG ATTAAGGAG	gc gcggccgc TTA ATTAAGCTTGTGC CCCAGTTTG	BamHI NotI	pTagBFP-C
pLAMP1-BFP	pBFPN1	GC GCTAGC gcc accATGGCGGCCC CCGGCAGCGCC	GC GGATCC GATAGTCTGGTA GCCTGCGTG	NheI BamHI	HeLa cDNA
pLAMP2-BFP	pBFPN1	GC GCTAGC gccacc ATGGTGTGCTTCC GCCTCTTCCCG	GC GGATCC AAATTGCTCATAT CCAGCATGATG	NheI BamHI	HeLa cDNA
pETM11SUMO3ΔNSTAT3sfGFP	pETM11 SUMO3 sfGFP	TTTTGGCCCACAG GGCCTTACCGGC CAGGCCAACCAC CCC	TTTTGGCCGATAT GGCCCATGGGGG AGGTAGCGCAC	SfiI	pCDNA3.1-DYK-STAT3
pMA-STAT3-gRNA	pMA-SpCas9-g1	guide sequences: 5'-GGCCATCCTGCT AAAATCAG-3'		BbsI	N/A
pAAV-EGFP-MMEJ	pAAV-MCS	ATAGCGGCCGCG CTCCAGGTACCG TGTGTCAAGCTG CTGTAGCTGATTC CATTGGGCCATC CTGCTAAAATCG GACTTGTACAGCT CGTCCATGCC	ATAGCGGCCGCT ACCCCTACTGGG ACTTGTGGTGAA CATATGCACACTT TGGTTTACAGTTG GGACCCCTGATG GTGAGCAAGGGC GAGGAG	NotI	ptfLC3
